# Diverse banana endophytes reveal potential genotype-driven community structure affected by domestication

**DOI:** 10.3389/fmicb.2026.1830341

**Published:** 2026-06-24

**Authors:** Shiva A. Aghdam, Amanda M. V. Brown

**Affiliations:** Department of Biological Sciences, Texas Tech University, Lubbock, TX, United States

**Keywords:** amplicon, domestication, endophyte, microbiome, *Musa*

## Abstract

Plant endophytic microbiomes play critical roles in plant health, productivity, and stress tolerance, however, their relationship with host genotype remains poorly understood. This study focused on endophytic microbiomes of six banana (*Musa* spp.) cultivars grown under shared environmental conditions to determine how genotype influences microbial diversity and structure. We used deep amplicon sequencing to investigate the endophytic microbiomes from above- and below-ground tissues of wild diploid cultivars *Musa balbisiana*, *M. balbisiana* “Thai Black”, and *M. textilis*, and domesticated triploid cultivars Dwarf Cavendish, Williams Hybrid, and hybrid FHIA-25, grown in sympatry. Across all samples, dominant genera included *Pseudomonas*, *Acinetobacter*, *Enterobacter*, *Devosia*, and *Rhizobium*, while 27.4% of ASVs were unclassified. Although many core taxa were shared, each cultivar and tissue harbored distinct low-abundance microbial taxa. Beta diversity analyses revealed that banana cultivar explained a small but significant proportion of community variation (Bray–Curtis *R*^2^ = 2.7%, *p* = 0.002; Weighted UniFrac *R*^2^ = 2.9%, *p* = 0.005), whereas tissue type and domestication contributed less to overall variation. PICRUSt2 predicted functional differences among endophytic communities across banana cultivars, with 49 pathways differing between wild and domesticated plants, including enrichment of lipid metabolism, biotin biosynthesis, and aromatic compound degradation in domesticated cultivars. However, because domestication status and ploidy differed among the selected cultivars, these effects could not be fully separated in the current study. Together, these results indicate that banana genotype influences endophytic microbiome composition and predicted function, although host genotype accounted for only a modest proportion of the observed variation, highlighting the importance of additional ecological and environmental factors in shaping plant-associated microbial communities.

## Introduction

Endophytic microbiome communities living within plants can significantly enhance plant health by promoting growth, stress tolerance, and disease resistance (Aly et al., 2010; Kusari et al., 2015; Maheshwari, 2017; Rodriguez et al., 2009; White and Torres, 2010). These microorganisms enhance nutrient acquisition, produce phytohormones, and support plant defense through antimicrobial activity and induction of systemic resistance (Brader et al., 2017; Hardoim et al., 2015). Recent studies highlight their role in stress tolerance, showing that endophytes can improve drought tolerance, reduce disease severity, and promote plant growth (Ali et al., 2026; Halo et al., 2026). Yet, to date, there is limited data on how a plant’s domestication status, ranging from wild to highly domesticated, impacts the plant’s endophytes (Barnes et al., 2025; Soldan et al., 2024). While the environment determines the pool of microbes available to colonize plant tissues, plants have many mechanisms to select (i.e., promote or restrict) the colonizing community. A few studies suggest that host evolutionary history and history of domestication can gradually change the microbial community composition, especially in the root system (Ferreira et al., 2023; Peiffer et al., 2013; Shenton et al., 2016). Simultaneously, selective plant breeding over the years tends to reduce host plant genetic diversity in modern plant cultivars (Li et al., 2013; Soldan et al., 2021; Soldan et al., 2024). Thus, modern cultivars may have lost some of the ability to attract beneficial microbes compared to their wild progenitors (Pérez-Jaramillo et al., 2016; Shenton et al., 2016). While these studies provide numerous examples of host domestication and cultivation-related changes in genotype and environmental factors impacting plant rhizosphere microbiomes, a significant gap remains in understanding how these factors impact endophytes, which, in turn, affect plant health.

Compared to soil and rhizosphere microbiomes, endophytic microbiomes remain poorly characterized due to their high diversity and low culturability (Afzal et al., 2019; Aghdam and Brown, 2021; Saikkonen et al., 2004; Tenguria et al., 2011). Nevertheless, previous studies suggest that both environment-driven factors (e.g., soil, climate) and host genetic factors may drive differences in communities associated with rhizosphere, root, and leaf (He et al., 2019; Leopold and Busby, 2020). Some data suggests endophyte communities tend to be consistent within host species (Bouffaud et al., 2014; Compant et al., 2019; Sessitsch et al., 2019), with host genetics influencing community structure (Bouffaud et al., 2014; Fitzpatrick et al., 2018; Wippel et al., 2021).

Thus, in the current study, we investigate endophyte diversity with a focus on banana (*Musa* spp.), testing the effects of host tissue and genotype with respect to domestication status and ploidy in cultivars with varying levels of disease resistance. Bananas serve as an ideal model for examining the effects of historical domestication and clonal propagation on plant microbial communities (Arvanitoyannis and Mavromatis, 2009; Mohandas and Ravishankar, 2016; Sardos et al., 2022). Particularly in the context of disease-protective endophytes, banana plants provide a rich system for study, due to the complex history of wild and human-driven hybridization, breeding, and cultivation, all of which have led to diverse patterns of disease resistance among the hundreds of extant *Musa* cultivars. Across wild and cultivated *Musa* spp., cultivars differ widely in their responses to diseases, ranging from high susceptibility to strong resistance. Major threats include fungal diseases such as *Fusarium* wilt, and Black Sigatoka, bacterial diseases such as *Xanthomonas* wilt, viral diseases including Banana bunchy top and Banana streak viruses, and also nematode damage by *Radopholus similis* (burrowing nematode) (Drenth and Kema, 2021; Esguera et al., 2024; Grajales-Amorocho et al., 2024; Lantican et al., 2023; Musabyemungu et al., 2025; Soares et al., 2021; Torres-Asuaje et al., 2023; Tripathi et al., 2008).

Evidence from various plants suggests that wild relatives often have greater disease resistance (Heslop-Harrison and Schwarzacher, 2007; Pegg et al., 2019; Ploetz, 2009), and given the importance of the microbiome to plant health, an important question is how endophytes may contribute to these differences in disease resistance. Therefore, in the current study, we address this gap by analyzing the diversity of endophytic microbes across different banana cultivars with distinct genetic backgrounds, including both wild and domesticated species of banana (*Musa* spp.), considering how differences in endophytes relate to each cultivar’s disease resistance profile. To control the various other factors that could shape endophytic community recruitment, all samples were grown in the same soil under the same conditions exposed to the same microbiota. We include wild diploid seed-producing plants (*Musa balbisiana*, *M. balbisiana* “Thai Black”, and *M. textilis*) and domesticated triploid seedless (parthenocarpic) plants (Dwarf Cavendish, Williams Hybrid, and hybrid FHIA-25). In this study, domestication status and ploidy are linked, as wild genotypes are diploid and domesticated cultivars are mostly triploid, so their effects cannot be fully separated. On the basis of previous studies and recent shotgun metapangenomics data (Singh et al., 2023), we predicted that wild banana cultivars (generally more disease resistant) would have more diverse bacterial endophytes than domesticated cultivars (generally less disease resistant), and that wild banana endophyte communities may display more disease-protective functions. Using 16S rRNA sequencing, we show that banana endophytic communities differ significantly among cultivars. These genotype-associated shifts extend to predicted functional potential, suggesting that host genetic background shapes both microbial composition and function.

## Methods

### Banana plant collection and surface sterilization

Endophytic communities from six banana (*Musa* spp.) cultivars representing distinct genotypes were collected from a small farm in Homestead, Florida, United States (25.4687° N, 80.4776° W). The collection included two triploid AAA cultivars (Dwarf Cavendish, *n* = 4 and Williams Hybrid, *n* = 4), two diploid B genome (*Musa balbisiana*, *n* = 4, and “Thai Black”, *n* = 4), a triploid AAB cooking plantain (FHIA-25, *n* = 3), and one other diploid species (*Musa textilis*, *n* = 1) (Supplementary Table S1). Young “suckers” (early-stage vegetative offshoots approximately 70–90 cm tall) were collected with each individual providing both above (leaf and stems) and below-ground (root/corm) tissues. Samples were collected during the 2019 and 2020 growing seasons. All plants were grown on the same farm under similar environmental conditions. Information on soil physicochemical properties and cropping history was not available for the sampling site and therefore was not included in this study. The relative resistance of these plants to various diseases, based on past studies, is shown in Supplementary Table S2. We collected up to four replicates of each cultivar, with above-ground and below-ground tissues processed separately from each plant, resulting in two tissue samples per individual. To remove external microbiota, plants were washed with tap water for 10 min, then tissues were surface sterilized following standard protocols (Refaei et al., 2011) by immersing in 70% ethanol for 1 min, 2.5% sodium hypochlorite solution for 3 min, 70% ethanol for 1 min, and then rinsing three times with sterile distilled water. Samples from both years were processed using the same protocols and analyzed together.

### Endophytic microbiome cell enrichment extraction protocol

Although amplicon sequencing can be performed directly from whole plant tissue DNA using standard extraction kits, we used a modified protocol designed to process a larger amount of starting plant tissue per sample. For this, we began with a modified microbiome cell enrichment protocol based on a published study (Singh et al., 2023) to remove a significant portion of banana cells. Briefly, this protocol breaks plant tissues mechanically and separates the microbes by a series of mesh filtrations followed by gradient centrifugation in Nycodenz^®^ (CosmoBioUSA: Catalog No. AXS-1002424). Specifically, 100 g wet weight of each plant sample was homogenized in a sterilized blender with 400 mL of ice-cold BCE buffer containing 50 mM Tris–HCl (pH 7.5), 1% Triton X-100 and 2 mM *β*-mercaptoethanol. Nycodenz^®^ solution was prepared at 0.8 g/mL by dissolving 3.2 g Nycodenz in 4 mL of 50 mM Tris–HCl buffer (pH 7.5) per sample.

### DNA extraction, and 16S rRNA amplicon data generation

DNA was isolated from the enriched microbiome layer using the QIAGEN DNeasy Blood & Tissue Kit (Valencia, CA) following the manufacturer’s directions. DNA was checked for quantity and quality on the NanoDrop™ spectrophotometer, then DNA was used for PCR. PCR was performed to produce amplicons using published universal bacterial primers for the 16S rRNA gene: 16S-341F (CCTACGGGAGGCAGCAG) and 806R (GGACTACHVGGGTWTCTAAT) targeting the V3–V4 region (Caporaso et al., 2012; Muyzer et al., 1993). PCR was performed with the heated lid set to 105 °C using the following thermal cycler conditions: initial denaturation at 95 °C for 5 min; 35 cycles of denaturation at 94 °C for 45 s, annealing at 56 °C for 45 s, and extension at 72 °C for 1 min; followed by a final extension at 72 °C for 10 min. PCR negative controls were included during amplification. PCR product size was checked via gel electrophoresis, and quantified with a NanoDrop™ spectrophotometer, then products were purified using Agencourt AMPure XP beads with a PCR product: nuclease-free water: bead ratio of 1:1:1.8. Purified products were quantified again using a NanoDrop™ spectrophotometer, then 200 ng of each product was used for amplicon library preparation using the QIAseq 1-Step Amplicon Library Kit (96) (Qiagen, United States) to create multiplexed barcoded libraries following the manufacturer’s directions. Libraries were quantified using the Agilent Tapestation 2,200, normalized, pooled, and then sequenced together in a single run on the Illumina MiSeq platform run with 2 × 250 bp paired-end sequencing at Genewiz (NJ, United States).

### Sequence analysis using QIIME2 and statistical tests

Bioinformatic analysis was conducted using the microbiome bioinformatics pipelines in QIIME2 (Quantitative Insights Into Microbial Ecology) version 2021.11 and later version 2024.2 (Dong et al., 2018; McKnight et al., 2020; Wallace et al., 2018). Quality filtering, merging, and denoising of sequences was performed using the DADA2 algorithm through the q2-dada2 plugin in QIIME2 (Callahan et al., 2016). DADA2 joined paired-end reads and then implemented a quality-aware correcting model for amplicon data that denoises sequences, removed chimeras and residual PhiX reads, dereplicates DNA reads, and calls amplicon sequence variants (ASVs). Sequences were truncated to 245 bp based on inspection of demultiplexed read quality profiles to retain high-quality bases (Q > 30) while maintaining sufficient overlap for paired-end merging in DADA2. The alignment of the remaining sequences was performed with MAFFT (Katoh and Standley, 2013), and a phylogeny was constructed from these sequences using the QIIME2 FastTree plugin (Price et al., 2010). Sequences classified as chloroplast, mitochondrial, archaeal, or eukaryotic/plant-derived were removed using QIIME2 through command line. The 132 release of the SILVA 16S rRNA database was used for taxonomic classification. Above-ground and below-ground tissues were initially analyzed separately for diversity analyses. However, because both tissue types originated from the same individual plants and cultivar replication was limited (≤4 plants per cultivar), tissue-level comparisons were not fully independent. Therefore, tissues were combined in selected downstream analyses to evaluate overall cultivar-associated microbiome patterns. To normalize sequencing depth across samples, we used the SRS (Scaling with Ranked Subsampling) method in q2-srs to adjust all samples have the same sequencing depth (Heidrich et al., 2021). Alpha diversity on normalized reads was compared in QIIME2 with q2 diversity alpha-group-significance using Shannon diversity, Chao1 richness and Faith phylogenetic diversity (Faith PD) vectors. The Kruskal–Wallis H-test (with FDR correction) was used to determine whether alpha diversity differed significantly among the groups and a significance level of *p*-value <0.05 was used as the threshold (Lin and Peddada, 2020). Beta diversity of the normalized data set was determined via PERMANOVA in the q2 diversity beta-group-significance plugin using the Bray-Curtis, Weighted and Unweighted UniFrac distance matrix (Anderson et al., 2006; Soininen et al., 2007). Multivariate ANOVA based on similarity tests (Adonis) were conducted using the Bray-Curtis, Weighted and Unweighted UniFrac distance matrices. These tests were performed to evaluate the relative influence of banana cultivar, domestication status, and tissue types on changes in microbial communities, determining which factors significantly impact the composition of these communities. Amplicon sequence variant (ASV) counts were grouped at the genus or family level. To allow CLR transformation of count data containing zeros, a pseudocount of 1 was added to all genus- and family-level counts before transformation. This value was selected as the smallest possible non-zero count to minimize distortion of relative abundance patterns while avoiding undefined log values. Counts were then converted to proportions and transformed using the centered log-ratio (CLR) method (McDonald et al., 2018; Wallen, 2021; Weiss et al., 2017). Differences in taxon abundance among cultivars were tested on the CLR-transformed data using Kruskal–Wallis tests with FDR correction, followed by Dunn’s post-hoc pairwise comparison tests, also adjusted for multiple testing in the rstatix package in R (Hill Janet and Albert Arianne, 2019; Lloréns-Rico et al., 2021). Analysis of Composition of Microbiomes with Bias Correction (ANCOM-BC) was used for differential abundance analysis (Lin and Peddada, 2020). The correction for multiple testing was performed using the Holm-Bonferroni method and a significance level of adjusted *p*-value <0.05 was used as the threshold (Heidrich et al., 2021). The alpha diversity plots were created using GraphPad Prism v. 10 (La Jolla, CA, United States) based on the outputs from QIIME2 to visualize the results. Additionally, ANCOM-BC, and PCoA plots of all indices for beta diversity were generated using ggplot2 R package (v. 4.3.1).

### Predicting the functional potential of the microbiome

PICRUSt2 (Phylogenetic Investigation of Communities by Reconstruction of Unobserved States) was used to predict the functional composition from metabarcoding amplicon 16S rRNA sequence data. The metabolic pathways were identified by using the q2-picrust2 plugin from QIIME2 v.2021.11. Alpha and beta diversity analyses based on the inferred functional profile were performed using the q2-core-metrics plugin. Metabolic pathways were predicted in the MetaCyc database using gene abundance predictions and PICRUSt2 default parameters. Differentially abundant metabolic pathways were analyzed using DESeq2 with an adjusted p-value cutoff of 0.05 (Love et al., 2014). The results were visualized using the “ggplot2” package v. 4.3.1 in R.

## Results

### Sequencing outputs and richness of endophytic bacteria

A total of 7,683,094 paired-end reads were obtained after demultiplexing, with per-sample read counts ranging from 54,671 to 1,406,725 reads. After quality filtering, merging, and removal of non-bacterial sequences, 4,897,553 high-quality reads remained for downstream analyses, ranging from 14,370 to 382,858 reads per sample. Rarefaction curves showed that increasing sequencing depth resulted in only small increases in detected ASVs for most samples (Supplementary Table S3; Supplementary Figure S1). A total of 13,852 unique amplicon sequence variants (ASVs) were detected across samples, with 27.24% of detected ASVs remaining unclassified across all cultivars and tissues (Supplementary Figure S2).

### Predominant endophytic bacteria among banana cultivars

**Figure 1:**
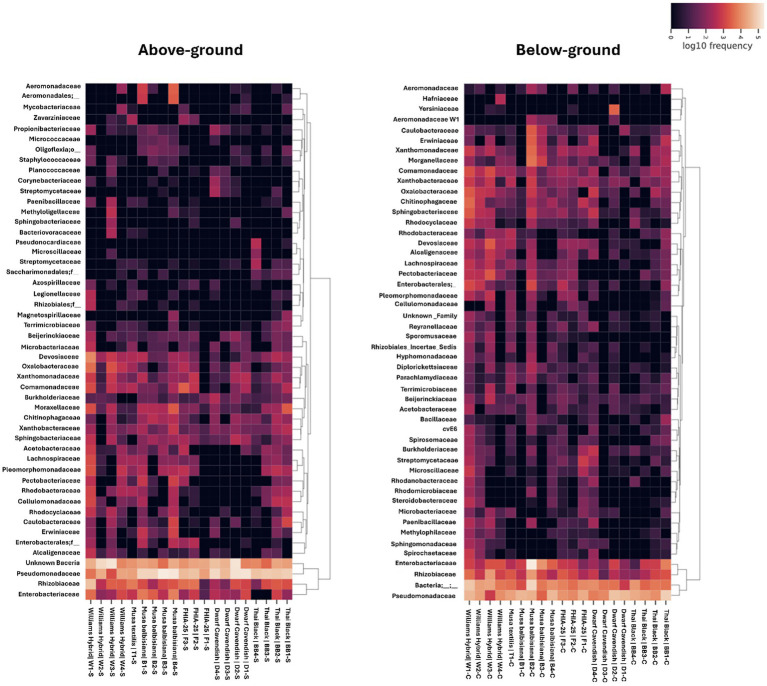
Heatmap showing hierarchical clustering of the top 50 most abundant endophytic bacterial families detected in above-ground and below-ground tissues across six banana cultivars. Samples are shown on the x-axis and bacterial families on the y-axis. Relative abundance values were log10-transformed prior to visualization. Color intensity represents the relative abundance of each bacterial family across samples, with lighter colors indicating higher abundance. Hierarchical clustering was performed based on similarity in bacterial family abundance profiles among samples and taxa.

Across all samples, endophytic bacterial ASVs were dominated by phyla Proteobacteria, Bacteroidetes, Verrucomicrobiota, Firmicutes, Actinobacteriota, Myxococcota, Bdellovibrionota, Patescibacteria, and Acidobacteriota (Supplementary Figure S2). At the family level, ASVs were dominated by *Pseudomonadaceae* (48.07%), *Enterobacteriaceae* (13.08%), and *Rhizobiaceae* (4.1%), with family-level community composition differing among banana cultivars (Figure 1; Supplementary Table S4). Dunn’s post-hoc pairwise tests following Kruskal-Wallis analysis revealed several significant differences in bacterial family abundances among cultivars. For instance, *Pseudomonadaceae* was less abundant in *M. balbisiana* compared to Williams Hybrid (p-adj = 0.01). Similarly, in Dwarf Cavendish, *Enterobacteriaceae* was significantly more abundant than in *M. balbisiana* (p-adj = 0.02). *Comamonadaceae* was more abundant in Dwarf Cavendish compared to Williams Hybrid (p-adj = 0.02), while *Devosiaceae* abundance was higher in Thai Black and Dwarf Cavendish compared to Williams Hybrid (p-adj = 0.03 and 0.0001, respectively) (Supplementary Table S5; Supplementary Figure S3).

**Figure 2:**
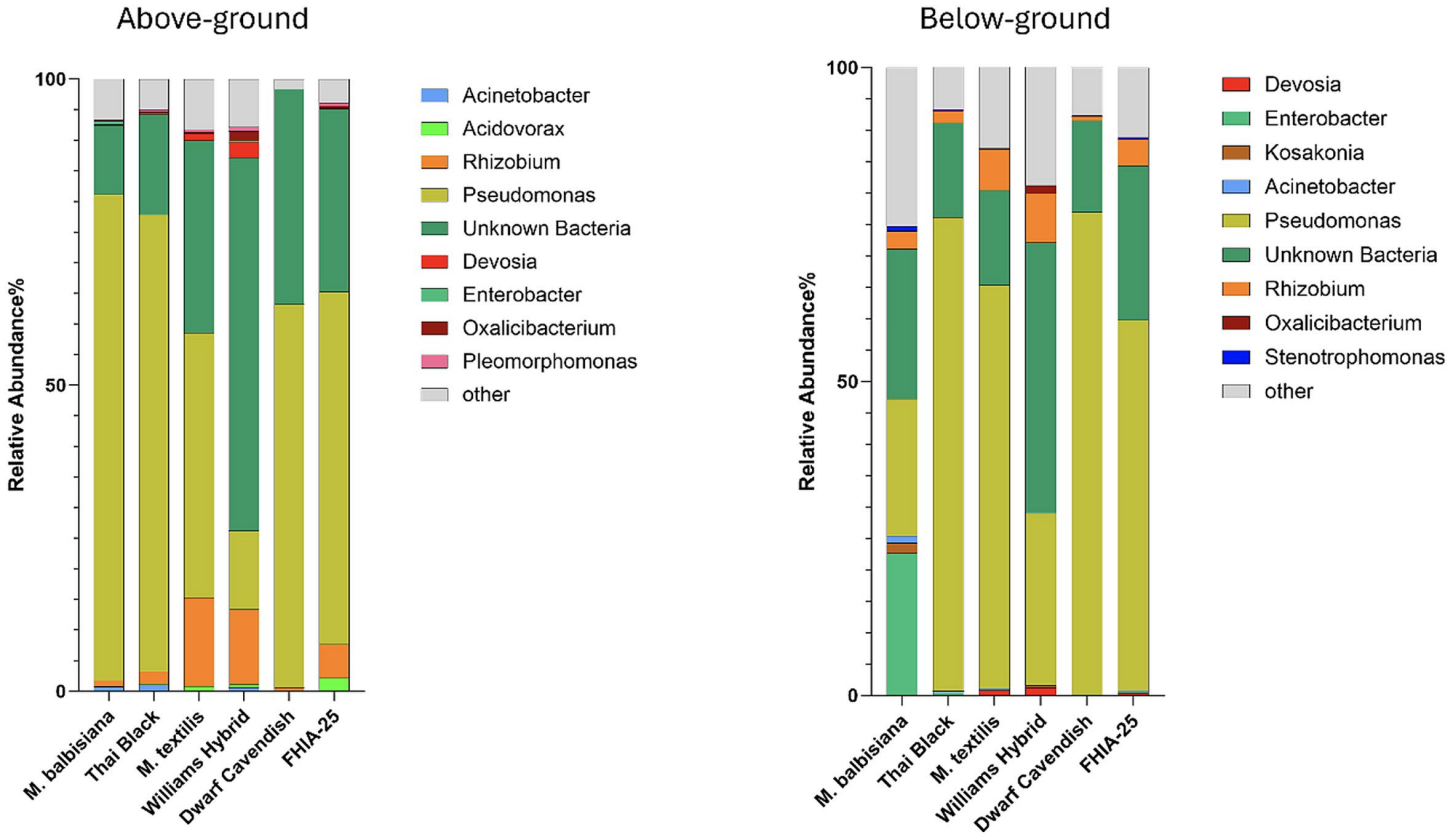
Relative abundance of dominant endophytic bacterial genera across six banana cultivars in above-ground and below-ground tissues. Stacked bar plots show the mean proportional abundance of the most abundant bacterial genera within each cultivar. For visualization, the eight most abundant genera and the category “Unknown Bacteria” are shown separately, while all remaining genera were grouped as “Other.” Relative abundance values are shown as percentages of total genus-level bacterial reads within each cultivar and tissue type.

**Figure 3:**
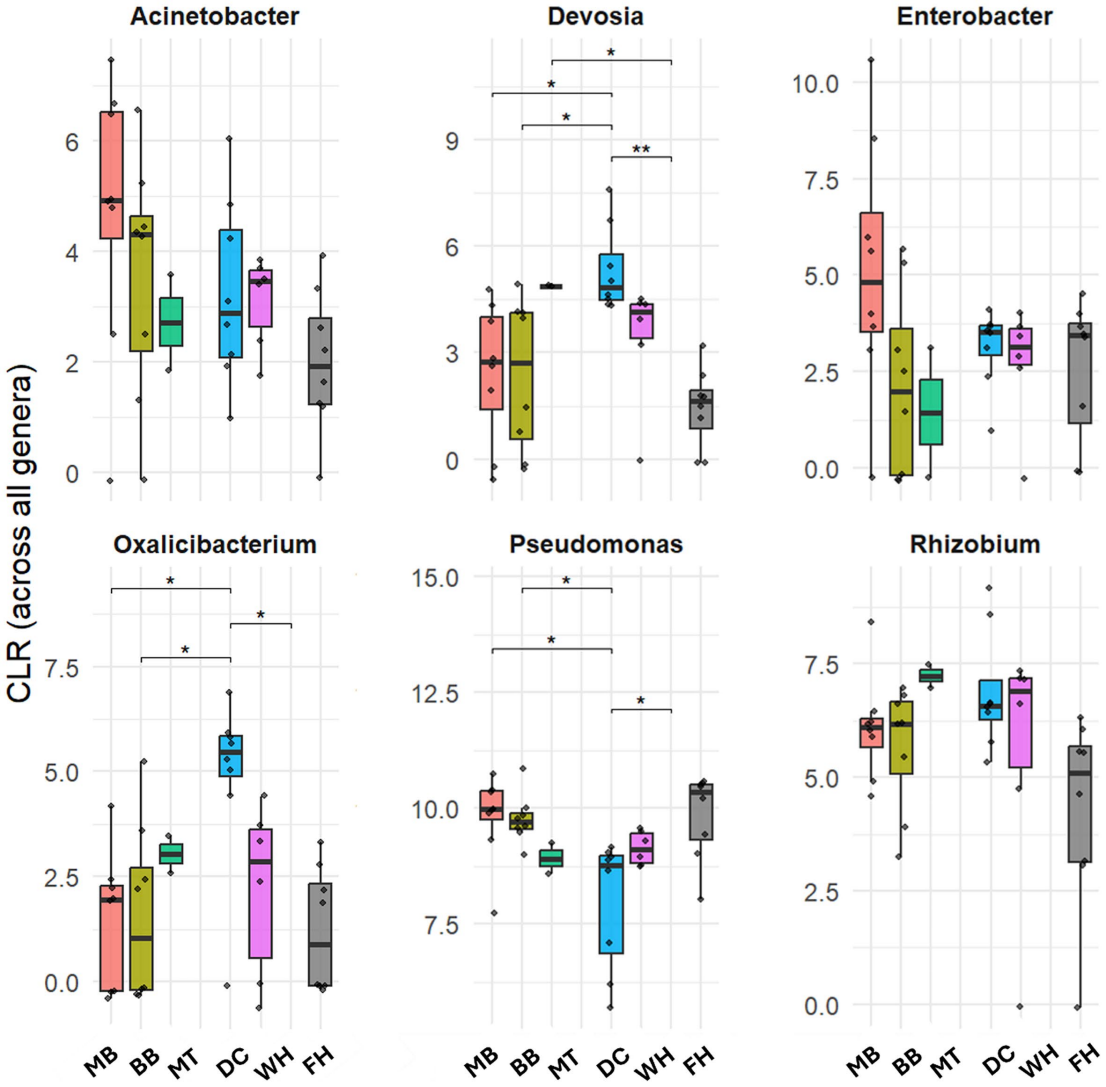
Centered log-ratio (CLR)–transformed abundances of six most abundant bacterial genera across banana cultivars. Boxplots show genus-level CLR values for *Acinetobacter*, *Devosia*, *Enterobacter*, *Oxalicibacterium*, *Pseudomonas*, and *Rhizobium* across the six cultivars (MB = *Musa balbisiana*; BB = Thai Black; MT = *Musa textilis*; DC = Dwarf Cavendish; WH = Williams Hybrid; FH = FHIA-25). Each point represents an individual sample. Boxes show the interquartile range, center lines indicate medians, and whiskers show the data range. Asterisks indicate significant differences among cultivars based on Kruskal–Wallis tests followed by Dunn’s post-hoc pairwise comparisons (FDR-corrected): *p* < 0.05 (*), *p* < 0.01 (**).

At the genus level, the most abundant and widespread taxa across all cultivars were *Pseudomonas*, *Acinetobacter*, *Enterobacter*, *Devosia*, *Rhizobium*, and *Oxalicibacterium* (Figure 2). Dunn’s post-hoc comparisons revealed multiple significant differences in bacterial genera among banana cultivars (Figure 3; Supplementary Table S6). Notably, *Devosia* was significantly more abundant in Dwarf Cavendish compared to Williams Hybrid (p-adj = 0.001) and also higher in Thai Black (p-adj = 0.01) and *M. balbisiana* (p-adj = 0.01) compared to Williams Hybrid. In addition, *Oxalicibacterium* showed higher relative abundance in Thai Black (p-adj = 0.01), Dwarf Cavendish (p-adj = 0.01), and *M. balbisiana* (p-adj = 0.01) compared to Williams Hybrid. On the other hand, *Pseudomonas* showed lower abundance in *M. balbisiana* (p-adj = 0.01) and Thai Black (p-adj = 0.01) compared to Williams Hybrid. Other genera showing significant cultivar-specific differences included *Acidovorax*, *Raoultella*, *Lachnotalea*, *Sphingopyxis*, *Kosakonia*, *Pectobacterium*, and *Xylella* (all p-adj < 0.05) (Figure 3; Supplementary Table S6).

### Endophytic bacterial community composition and diversity differences across banana tissues and cultivars

**Figure 4:**
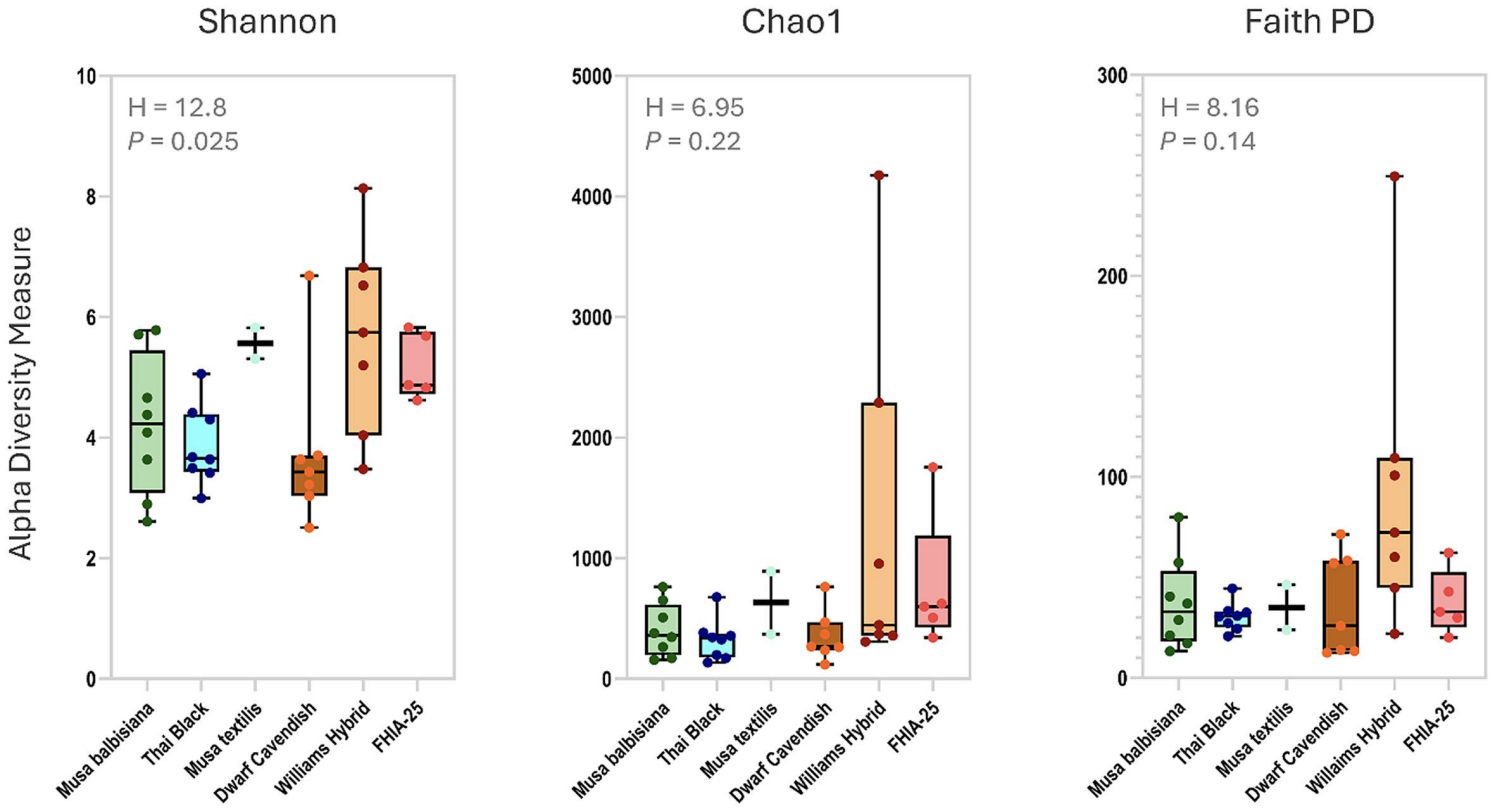
Alpha diversity of endophytic bacterial communities across banana cultivars. Boxplots show Shannon diversity, Chao1 richness, and Faith’s phylogenetic diversity (Faith PD) for bacterial communities across six banana cultivars. Each point represents an individual sample. Boxes show the interquartile range, center lines indicate medians, and whiskers show the data range. Overall differences among cultivars were tested using Kruskal–Wallis tests, with test statistics and *p*-values shown within each panel.

Shannon diversity differed among the six banana cultivars (Kruskal–Wallis H = 12.8, *p* = 0.02), with Williams Hybrid showing the highest diversity (5.70) and Dwarf Cavendish the lowest (3.27). The remaining cultivars ranked as follows: *M. textilis* (5.56), FHIA-25 (5.17), *M. balbisiana* (4.22), and Thai Black (3.87). Although overall differences were significant, no pairwise comparisons remained significant after FDR correction (Figure 4; Supplementary Table S7A). Across tissue types, Shannon H was not significantly different (Kruskal–Wallis H = 2.94, *p* = 0.08; Supplementary Table S7A). Chao1 richness showed no significant difference among cultivars (Kruskal–Wallis H = 6.96, *p* = 0.22), although average values varied, with Williams Hybrid showing high richness (1271.90), followed by FHIA-25 (765.15) and *M. textilis* (631.03, Figure 4; Supplementary Table S7B). However, Chao1 richness differed significantly between above- and below-ground tissues across cultivars (Kruskal–Wallis H = 5.2, *p* = 0.02; Supplementary Table S7B). Furthermore, phylogenetic diversity (Faith’s PD) showed no significant differences among cultivars (Kruskal–Wallis H = 8.17, *p* = 0.15; Figure 4; Supplementary Table S7C), although average Faith’s PD value varied, with Williams Hybrid showing high diversity (94.18), and the lowest observed in Thai Black (30.56).

**Figure 5:**
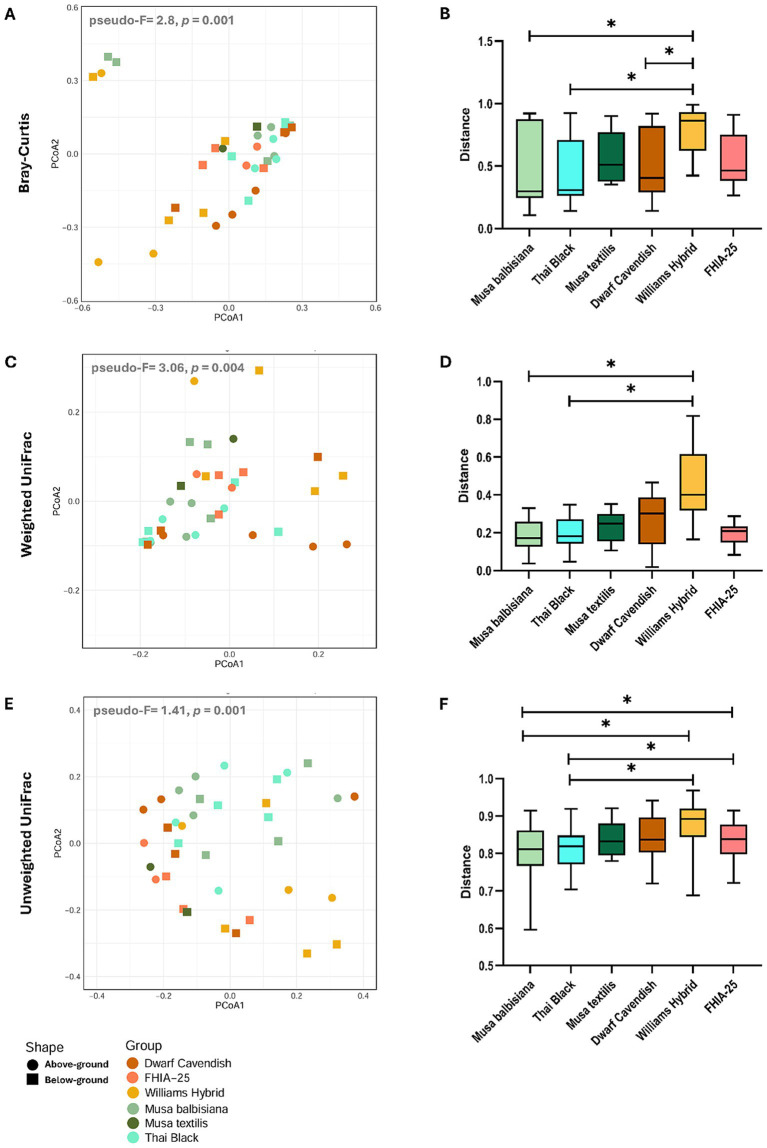
Beta diversity of endophytic bacterial communities among banana plants shown by PCoA plots (left) and box-and-whisker plots (right) for **(A,B)** Bray–Curtis dissimilarity, **(C,D)** Weighted UniFrac, **(E,F)** Unweighted UniFrac, with results of overall PERMANOVA tests shown in left plots PCoA panels, with pseudo-F and *p*-values indicated. Pairwise PERMANOVA results are shown as asterisks for statistically significant comparisons after multiple-testing correction. Points in the PCoA plots represent individual samples, with colors indicating cultivar and shapes indicating tissue type.

Beta diversity analyses showed that community composition differed significantly among banana cultivars based on Bray–Curtis and UniFrac distance metrics (PERMANOVA; Figure 5; Supplementary Table S8). Bray–Curtis dissimilarity revealed significant variation in microbial community structure among cultivars (Pseudo-*F* = 2.8, *p* = 0.001). Similarly, both Unweighted UniFrac (Pseudo-*F* = 1.41, *p* = 0.001) and Weighted UniFrac (Pseudo-*F* = 3.06, *p* = 0.004) showed significant differences in community composition across cultivars (Supplementary Table S8A). Pairwise PERMANOVA comparisons indicated that based on Bray–Curtis dissimilarity, significant differences were observed between Williams Hybrid and Thai Black (Pseudo-*F* = 6.34, q = 0.015), Williams Hybrid and Dwarf Cavendish (Pseudo-*F* = 3.52, q = 0.04), and Williams Hybrid and *M. balbisiana* (Pseudo-*F* = 3.83, q = 0.015) (Figure 5B; Supplementary Table S8B). Weighted UniFrac analyses also showed distinct community compositions between Williams Hybrid and Thai Black (Pseudo-*F* = 6.04, q = 0.015) and between Williams Hybrid and *M. balbisiana* (Pseudo-*F* = 6.41, q = 0.015) (Figure 5D; Supplementary Table S8B). Finally, Unweighted UniFrac comparisons revealed additional significant differences between Thai Black and FHIA-25 (Pseudo-*F* = 1.59, q = 0.03), Thai Black and Williams Hybrid (Pseudo-*F* = 1.72, q = 0.03), *M. balbisiana* and Dwarf Cavendish (Pseudo-*F* = 1.83, q = 0.03), and *M. balbisiana* and Williams Hybrid (Pseudo-*F* = 1.61, q = 0.03) (Figure 5F; Supplementary Table S8B).

To further evaluate the influence of host factors, multivariate Adonis tests were performed to examine how banana cultivar, tissue type, and domestication status affected bacterial community composition (Supplementary Table S8C). The analysis showed that cultivar had a significant effect across all distance metrics: Bray–Curtis (*R*^2^ = 2.7%, *p* = 0.002), Unweighted UniFrac (*R*^2^ = 1.4%, *p* = 0.013), and Weighted UniFrac (*R*^2^ = 2.9%, *p* = 0.005). Domestication (ploidy) also showed a significant effect on Bray-Curtis (*R*^2^ = 3.6%, *p* = 0.004), Unweighted UniFrac distances (*R*^2^ = 1.8%, *p* = 0.002), and Weighted UniFrac (*R*^2^ = 6.17%, *p* = 0.002). Tissue type influenced community composition only under Unweighted UniFrac (*R*^2^ = 1.6%, *p* = 0.005), and no significant effects of tissue were observed under Bray–Curtis or Weighted UniFrac metrics.

### Differential abundance of bacterial families among banana cultivars using ANCOM-BC

**Figure 6:**
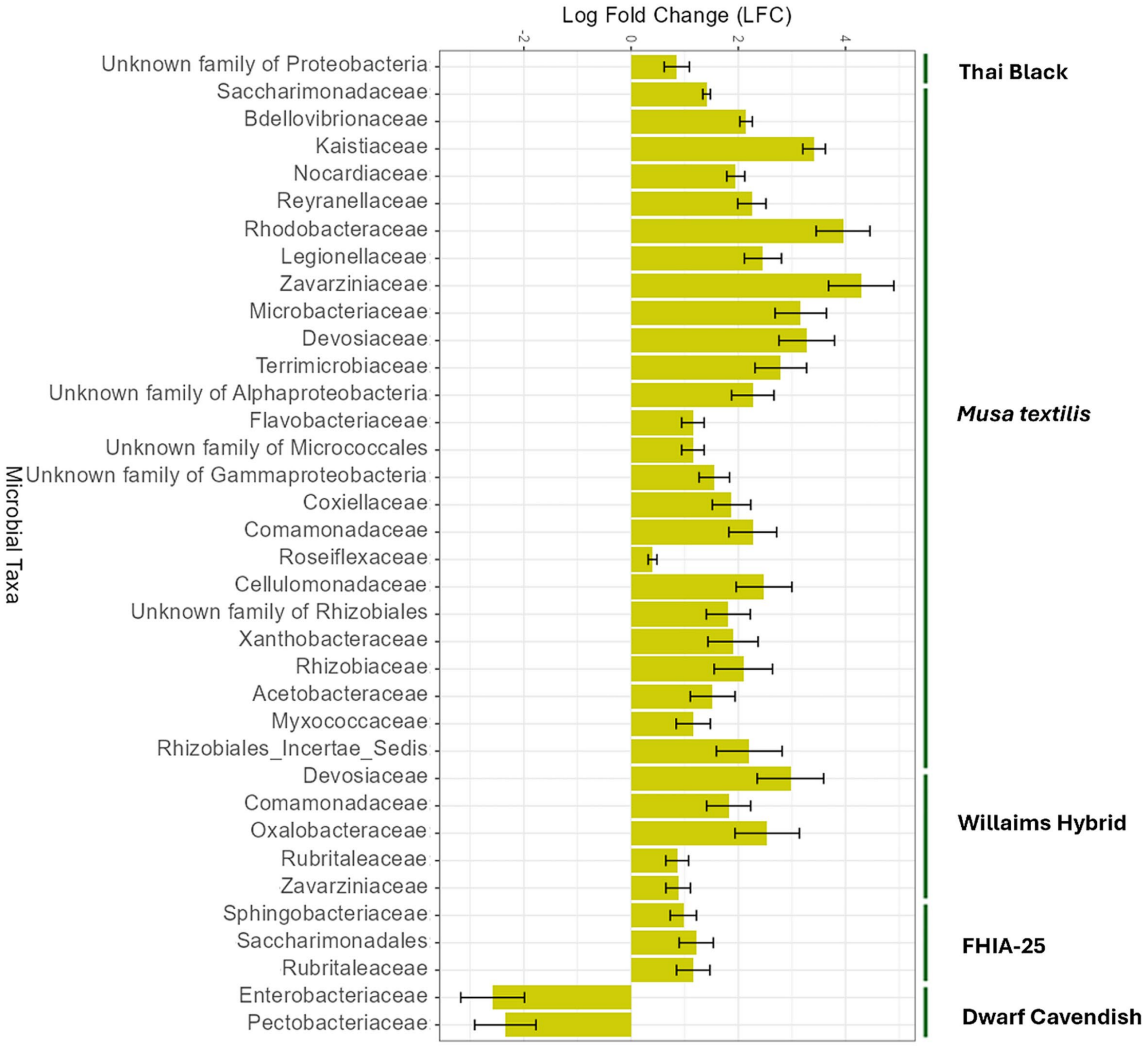
Differential abundance analysis of microbial taxa across various banana cultivars relative to *Musa balbisiana*, as indicated by ANCOM-BC. Each bar represents the log fold change in abundance of specific bacterial families with standard error by sample type within cultivars *Musa textilis*, FHIA-25, Williams Hybrid, Dwarf Cavendish, and Thai Black. Bars show log fold change values, with error bars representing standard errors. Positive values (right) depict enrichment, while negative values (left) depict depletion of taxa compared to *Musa balbisiana.*

Due to the limited number of shared genera, ANCOM-BC was applied at the family level, which provided sufficient data for accurate detection of differential abundance among cultivars. Our analysis using ANCOM-BC showed significant differences in the abundance of specific microbial taxa at the family level associated with different cultivars (Figure 6; Supplementary Table S9). Compared to *M. balbisiana*, *M. textilis* showed a diverse set of significantly increased bacterial families such as: *Zavarziniaceae* (lfc = 4.2, q < 0.01), *Rhodobacteraceae* (lfc = 3.95, q < 0.01), and *Kaistiaceae* (lfc = 3.41, q < 0.01), *Devosiaceae* (lfc = 3.27, q < 0.01), *Microbacteriaceae* (lfc = 3.1, q < 0.01), *Terrimicrobiaceae* (lfc = 2.7, q < 0.01), *Legionellaceae* (lfc = 2.45, q < 0.01), and *Comamonadaceae* (lfc = 2.2, q < 0.01). Compared to *M. balbisiana*, Thai Black showed a higher abundance of an unclassified family within Proteobacteria (lfc = 0.85, q < 0.05). Dwarf Cavendish had lower abundances of *Pectobacteriaceae* (lfc = −2.3, q < 0.01), and *Enterobacteriaceae* (lfc = −2.5, q < 0.01). FHIA-25 showed higher abundances of *Saccharimonadales* (lfc = 1.21, q < 0.05), *Rubritaleaceae* (lfc = 1.15, q < 0.05), and *Sphingobacteriaceae* (lfc = 0.97, q < 0.05). Williams Hybrid showed higher abundances of *Devosiaceae* (lfc = 2.97, q = 0.0002), *Oxalobacteraceae* (lfc = 2.5, q < 0.01), *Comamonadaceae* (lfc = 1.81, q < 0.01), *Rubritaleaceae* (lfc = 0.86, q < 0.01) and *Zavarziniaceae* (lfc = 0.87, q < 0.05) (Figure 6; Supplementary Table S9).

### Comparison of the effect of various banana cultivars on predicted functional capacity of the microbiome

**Figure 7:**
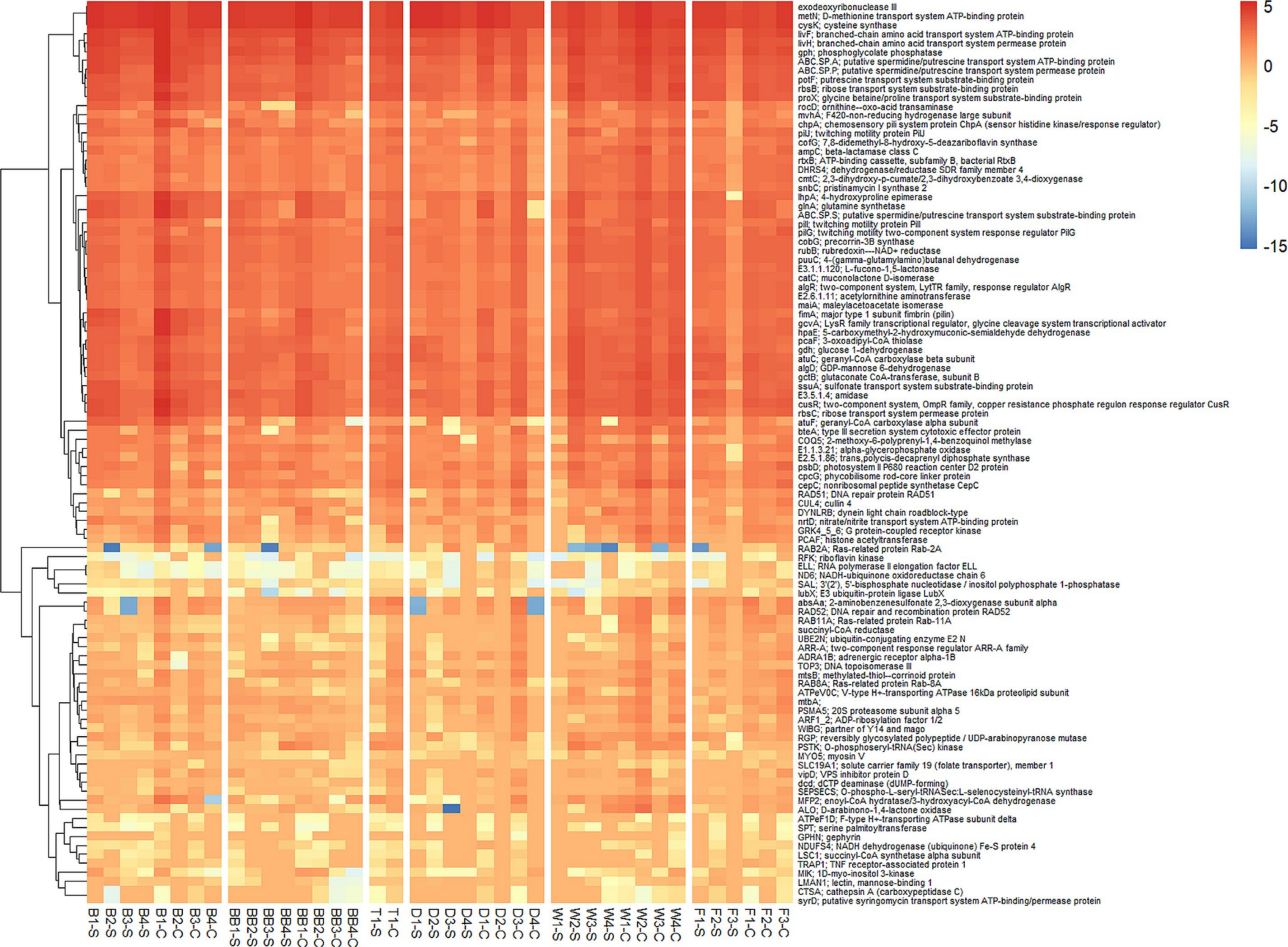
A cluster heatmap showing the relative abundance of different metabolic pathways between bacterial communities associated with various banana cultivars based on PICRUSt2 analysis. Rows represent individual predicted pathways clustered based on abundance, and columns represent samples categorized by banana cultivars and tissue type. Color intensity indicates log-transformed predicted pathway abundance, with red color representing higher abundance and blue colors representing lower abundance. Hierarchical clustering was performed based on similarity in predicted pathway abundance profiles.

Using PICRUSt2, we predicted the functional pathways associated with microbial communities across various banana cultivars, producing a heatmap (Figure 7) of the normalized log-transformed relative abundances of these predicted pathways across samples, highlighting distinct functional patterns. Although some key metabolic pathways, such as PHOSLIPSYN-PWY (phospholipid synthesis), the TCA cycle, and ARGSYN-PWY (arginine biosynthesis), were consistently represented across samples, other pathways differed among samples, as shown in the results of alpha and beta diversity analyses on microbial functions. Functional diversity differences, assessed with the Shannon diversity index, showed a statistically significant difference among the cultivars (Kruskal–Wallis H = 14.39, *p* = 0.013, Supplementary Table S10A). In contrast, the number of predicted functional pathways, reflecting microbial functional richness, showed no significant differences among the groups (Kruskal–Wallis H = 9.82, *p* = 0.08, Supplementary Table S10B).

**Figure 8:**
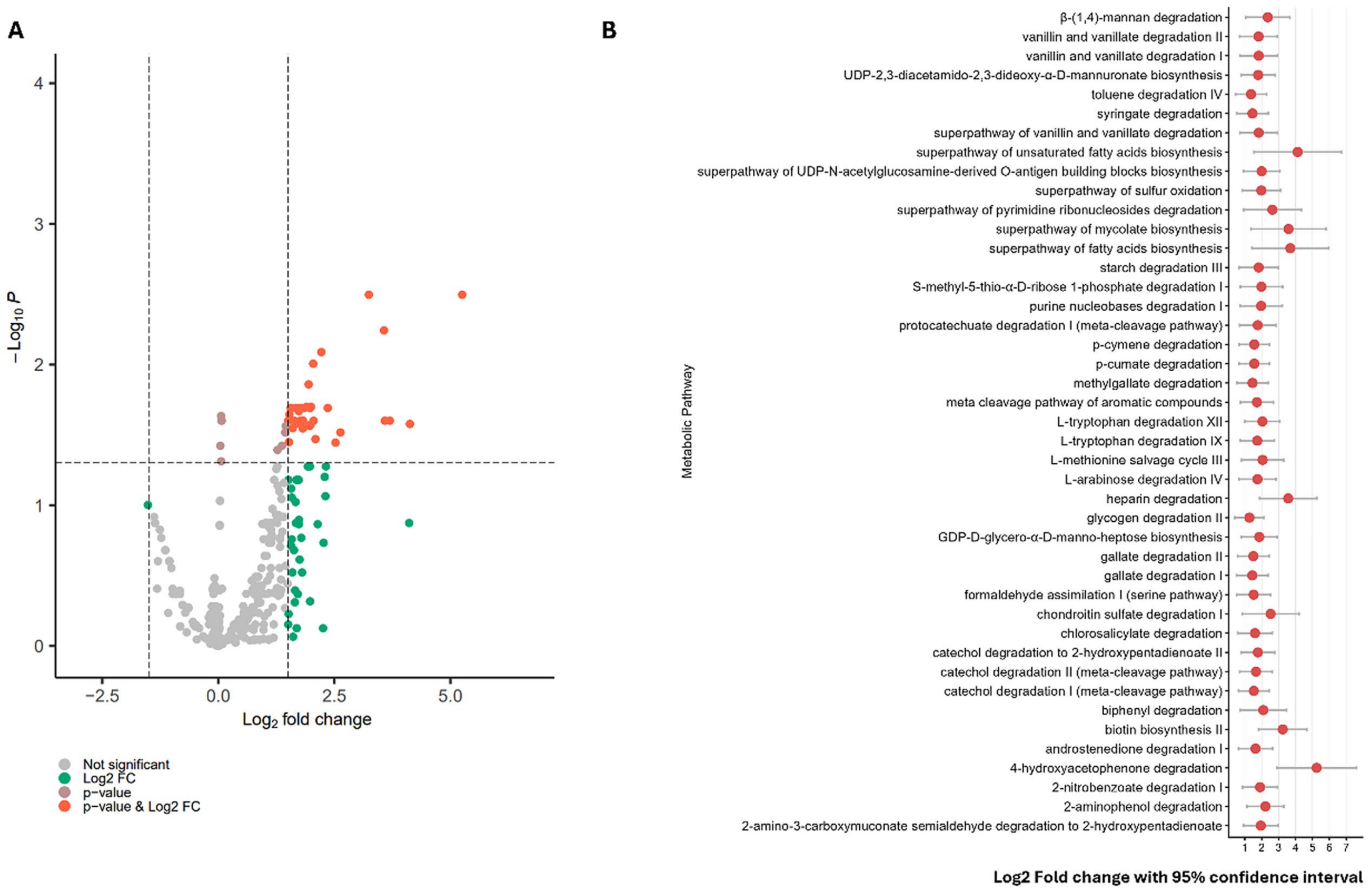
Analysis of predicted functional differences among banana endophyte communities. **(A)** Volcano plot showing the statistical significance and fold change of predicted MetaCyc metabolic pathways derived from PICRUSt2 analysis of 16S rRNA data comparing wild and domesticated cultivars. Gray dots represent non-significant changes, purple dots indicate pathways significant by *p*-value (not FDR-corrected), green dots show log2 fold changes (LFC) greater than 1, and red dots denote significant metabolites with q < 0.05. **(B)** predicted MetaCyc metabolic pathways from the microbiomes of domesticated cultivars, identified through PICRUSt2 analysis, are categorized by log2 fold change and q < 0.05 in comparison to wild plants. Error bars represent 95% confidence intervals. Because these results are based on PICRUSt2 predictions from 16S rRNA gene profiles, they represent predicted functional potential rather than directly measured microbial activity.

Functional beta diversity differences, assessed with Bray–Curtis dissimilarity and PERMANOVA tests suggested significant differences in predicted functions across the cultivars (Pseudo-*F* = 3.39, *p* = 0.013, Supplementary Table S10C). Specific tests of such functional differences, analyzed using DESeq2, identified differences in predicted metabolic pathways between wild and domestic cultivars. Specifically, among 490 tested pathways, 49 pathways showed significantly different abundance between the microbiomes of wild and domesticated plants (Figure 8A; Supplementary Table S11). Pathways such as 4-hydroxyacetophenone degradation (lfc = 5.2, p-adj = 0.003), the super pathway of unsaturated fatty acid biosynthesis (lfc = 5.2, p-adj = 0.003), biotin biosynthesis II (lfc = 0.72, p-adj = 0.003), and biphenyl degradation (lfc = 0.7, p-adj = 0.03) were enriched in domesticated plants. No metabolic pathways were significantly enriched in wild cultivars relative to domesticated cultivars (Figure 8B; Supplementary Table S11).

## Discussion

This study analyzed endophytic microbiome communities from wild and domesticated banana plant cultivars to infer how domestication status and associated differences in disease resistance, may impact community composition from above- and below-ground tissues. Given that all banana cultivars were grown in sympatry, with exposure to the same colonizing microbiota, this study effectively tested host-driven differences. Results showed that the endophytic microbiome community differed among hosts and between plant compartments, in a pattern associated with domestication level (wild vs. domesticated), ploidy, and known differences in disease resistance. These findings together suggest that *Musa* cultivars could drive beneficial endophytic microbiome recruitment according to their needs or evolved disease resistance.

We found the endophytic microbiome in below-ground banana tissues from domesticated triploid plants was more diverse than that in wild (diploid) plants, contrary to our prediction. The basis of our prediction was that healthier and more disease-resistant wild diploid hosts should retain the genetic capacity to recruit a richer array of beneficial host-associated microbes compared to domesticated cultivars that have lost genetic diversity in their own genomes. However, importantly, our study examined banana plants grown in a common small farm in Florida where the pool of available environmental endophytes may differ from those available in habitats where wild *Musa* spp. are endemic, such as Southern Asia. Thus, the endophyte pool in the source farm in our study may be enriched for species that colonize domesticated banana, given that the region in Florida has a history of banana cultivation – perhaps to the 16th century (Salas-Pascual and Cáceres-Lorenzo, 2022)—possibly explaining the increased diversity in domesticated plants. Alternatively, our findings of lower endophyte diversity in wild plants could be explained by arguments from the holobiont theory, recently discussed (Barnes et al., 2025), in which the sum of the plant and endophyte genomes is most important, such that the richer communities in domesticated banana are compensating for the plant’s own genome deficiencies. This idea is consistent with previous studies of rhizosphere microbes showing higher microbial diversity in domesticated plants compared to wild plants (Abdullaeva et al., 2021; Pérez-Jaramillo et al., 2016; Soldan et al., 2021; Soldan et al., 2024; Yue et al., 2023). Another explanation for our results could be that ploidy itself may influence how plants regulate endophytic colonization. Recent studies support this idea. For example, uncovering the reciprocal effects of plant polyploidy and the microbiome showed that genome duplication can change root microbiome composition and plant traits: neopolyploidy has been shown to alter microbial diversity, although the effects vary depending on the diploid genetic background (Anneberg et al., 2024). Triploid *Cannabis sativa* plants have been shown to harbor distinct endophytic communities compared to their diploid counterparts, suggesting that ploidy level can influence endophyte recruitment in crops (Srivastava et al., 2024). However, a limitation of this study is that domestication and ploidy are linked, as wild genotypes are diploid while domesticated cultivars are mostly triploid. Therefore, we cannot distinguish whether the observed differences are driven by domestication, ploidy, or their combined effects, and the results should be interpreted accordingly. In addition, all samples were collected from a single farm, meaning that the environmental microbial pool was shared across plants and may have influenced community composition. As a result, some of the observed differences among cultivars may reflect local environmental conditions rather than host-related factors alone. Together, these limitations emphasize the importance of future studies with expanded sampling, increased replication, and broader geographic coverage to validate and generalize these findings.

Our results showed the composition of bacterial communities associated with *Musa* spp. differed between above- and below-ground tissues, consistent with shotgun genomic data (Singh et al., 2023), but Adonis tests suggested the tissue effect was not as well-supported as the cultivar effect. Nevertheless, these banana plants hosted a wide range of endophytic bacterial communities across different tissues with most frequent representatives falling within the phyla *Actinobacteriota*, *Firmicutes*, *Gemmatimonadota*, *Nitrospirota*, and with *Proteobacteria*, consistent with previous studies (Alkhalili and Canbäck, 2018; Sachdev et al., 2010) and suggesting that these major groups of bacteria also play crucial roles in banana plant health and stress responses. Indeed, several abundant taxa were largely shared across all samples, as expected for plants grown in sympatry. Because all cultivars were grown under shared environmental conditions within the same farm, the available environmental microbial pool was likely similar across plants. However, using a single farm also limits how broadly these results can be generalized to banana plants grown under different environmental conditions. Nevertheless, differences among less dominant or tissue-specific taxa became evident when tissues were analyzed separately. Furthermore, a large proportion of leaf bacterial groups were shared with roots, suggesting inoculation from soil is the main source of the community within leaves, as has been observed previously (Wagner et al., 2016). However, when we compared the endophytic microbial communities of wild and domesticated banana plants with combined data from above- and below-ground parts of plants (i.e., tissues not separated in analysis), we found no differences in endophyte community using Weighted and Unweighted UniFrac metrics. This result may reflect the emphasis of UniFrac on dominant microbial taxa (Bolyen et al., 2019). The lack of statistically significant differences here may also be due to variance among replicates of each cultivar. Such variance is consistent with previous laboratory studies indicating that microbiome communities in below-ground tissues and the rhizosphere may vary due to intraspecific (inter-individual) genetic variation among plants (Bodenhausen et al., 2014; Bressan et al., 2009; Bulgarelli et al., 2012).

Beyond results comparing wild and domesticated cultivars, our results from individual cultivars showed significant differences for all Adonis tests, and most ASVs classified at the genus-level were specific to individual *Musa* cultivars, which is consistent with metagenomic data (Singh et al., 2023) and supports the idea of host genotype controlling endophyte colonization, as has been reported in other studies from various plant species (Bouffaud et al., 2014; Fitzpatrick et al., 2018; Regalado et al., 2020; Wippel et al., 2021). Specific effects of host genotype reported in the past have been diverse. For example, certain cereal crops have been shown to selectively recruit bacterial endophytes that enhance nutrient uptake (Bouffaud et al., 2014). Genetic variation in wild grass species can significantly shape root-associated fungal communities, potentially influencing drought resistance (Fitzpatrick et al., 2018). Distinct microbial communities have been associated with different grapevine genotypes, potentially contributing to pathogen resilience (Regalado et al., 2020). Furthermore, different legume genotypes have been observed to host unique rhizobial communities, which may affect nitrogen fixation efficiency (Wippel et al., 2021).

Notably, it appears that diversity among these banana endophyte communities across cultivars shows no particular pattern with respect to host plant genetic similarity (see host plant phylogeny in Figure 1 of Singh et al., 2023), i.e., endophytes from the more closely related cultivars Dwarf Cavendish and Williams Hybrid, or *M. balbisiana* and Thai Black, did not form obvious closely related clusters compared to less closely related FHIA-25 and *M. textilis*. This finding suggests that smaller genetic differences between cultivars can have big effects on endophytes. Clearly, small changes in banana genotypes can confer major changes in response to pathogenic microbes as seen by differences in disease resistance between Dwarf Cavendish and Williams Hybrid, or FHIA-25 and other hybrids (Chen et al., 2019; Dale et al., 2017; Fei et al., 2019; Hooks et al., 2009; Martínez-de la Parte et al., 2025; Thangavelu et al., 2025), so it might be expected that such genotype differences can have major impacts on many endophytic microbes. These large effects of small host differences may also explain the relatively high variance between replicate plants in our study.

Examining specific dominant genera in our study including *Pseudomonas*, *Acinetobacter*, *Enterobacter*, *Devosia*, and *Rhizobium*, we note that these have also been reported in previous studies of banana-associated microbiomes from various geographic regions such as Brazil, China, Costa Rica, the Canary Islands, India, Malaysia, Mexico, Nicaragua, Tanzania, and Uganda at various time and across different plant cultivars (Birt et al., 2022; Blomme et al., 2017; Junior et al., 2018; Karthik et al., 2017; Kaushal et al., 2020; Liu Y. et al., 2019; Nakkeeran et al., 2021; Ploetz, 2015). This consistent presence of these endophytes suggests they play important roles in banana plant health.

Regarding specific taxonomic differences, our observation of higher abundances of *Pseudomonas* in wild diploid cultivars compared to domesticated triploids was consistent with previous studies showing that wild genotypes may harbor more robust microbial defenses (Bajpai and Johri, 2018; De Smet et al., 2016; Ma et al., 2017) given that plant-associated *Pseudomonas* spp. often enhance plant health and act as effective biocontrol agents against fungal pathogens including *Fusarium oxysporum* (Bubici et al., 2019; Ghadamgahi et al., 2022; Prigigallo et al., 2022). The second most dominant genus found in our banana plants was *Acinetobacter*, which was previously recognized for its capabilities in nitrogen fixation, producing siderophores, and solubilizing minerals (Rahman et al., 2018; Sachdev et al., 2010). Additionally, *Rhizobium* spp. and *Enterobacter* spp. were found in higher abundance in our banana varieties. These taxa are known for their production of the auxin phytohormone indole-3-acetic acid, nitrogen fixation, and phosphate solubilization capabilities (Chi et al., 2005; Ranawat et al., 2021; Tewari and Sharma, 2020). These taxa may also impact various aspects of plant health and growth. Specifically, banana plants treated with strains of *Enterobacter* spp., *Pseudomonas* spp., and *Rhizobium* spp. have shown improved growth (Bajpai and Johri, 2018; Ghadamgahi et al., 2022; Kumawat et al., 2022; Macedo-Raygoza et al., 2019). However, the benefits of these bacteria may depend on the specific species and strain and further exploration is needed to determine the relative impacts of existing or augmented microbes in these groups (Abdelaziz et al., 2022; Birt et al., 2022; Bubici et al., 2019; Liu F. et al., 2019).

Furthermore, several bacterial families with significant cultivar-specific differences are known for contributing to plant health and stress tolerance. For example, *Devosiaceae* include taxa involved in nitrogen cycling, oxidative stress tolerance, and biofilm formation that can promote plant growth under challenging conditions (Lee et al., 2017; Shah et al., 2022). *Comamonadaceae* and *Oxalobacteraceae* are frequently enriched in the rhizosphere and endosphere of disease-resistant or stress-tolerant plants, where they contribute to nutrient turnover and suppression of soil-borne pathogens (Berg et al., 2014; Bulgarelli et al., 2013; Yu et al., 2021). Families such as *Microbacteriaceae* has been reported as common endophytes in banana and other tropical crops, with members capable of producing antifungal or plant-growth-promoting metabolites (Nakkeeran et al., 2021). Conversely, the reduced abundance of *Enterobacteriaceae* and *Pectobacteriaceae* in Dwarf Cavendish is notable, as these groups contain several opportunistic plant pathogens (Beltran-Garcia et al., 2021). Overall, these results suggest that differences among cultivars reflect both host-driven selection and ecological processes of microbial taxa with functions potentially linked to disease resistance and nutrient acquisition.

In addition to differences in bacterial composition, many pathways were enriched in domesticated cultivars, some of which appeared to be linked to microbial adaptation to the host environment or potential benefits to plant health. For example, PICRUSt2 analysis predicted enrichment of pathways associated with fatty acid and phospholipid biosynthesis that contribute to microbial membrane stability and biofilm formation, which can enhance colonization and stress tolerance within plant tissues (Mendes et al., 2013). The enrichment of biotin biosynthesis pathways suggests a potential role for microbial vitamin provisioning, a well-documented symbiotic function in plant-microbe systems (Gupta et al., 2020). Likewise, enrichment for aromatic compound degradation pathways, such as 4-hydroxyacetophenone and biphenyl degradation, may reflect microbial detoxification mechanisms or utilization of plant-derived phenolics, processes linked to the modulation of plant defense signaling (Venturi and Keel, 2016). However, these results are based on PICRUSt2 predictions from 16S rRNA data and therefore represent predicted functions rather than direct measurements. Together, these findings indicate that domesticated banana cultivars may favor microbial communities with enhanced metabolic versatility, particularly in lipid metabolism, stress mitigation, and xenobiotic processing, consistent with studies showing that domestication can alter microbial functional potentials toward more host-associated or nutrient-cycling roles (Pérez-Jaramillo et al., 2016; Yue et al., 2023).

In summary, this study suggested that banana cultivar tissue type, and domestication status collectively may influence the composition and predicted functions of the endophytic bacterial microbiome. While cultivar explained a statistically significant portion of the variation in community composition, the relatively low effect sizes (*R*^2^ ≈ 1–6%) show that host genotype accounts for only a limited proportion of total microbiome variation, suggesting that additional ecological and environmental factors also contribute to community assembly (Fitzpatrick et al., 2018; Wippel et al., 2021). We observed distinct differences in endophytic bacterial community composition and inferred function between various banana cultivars. Beta-diversity and multivariate analyses confirmed significant differences in community structure across cultivars, indicating strong host genotype effects, with additional but smaller influences of tissue type and domestication. Additionally, we found 27.24% of ASVs belonged to “unknown bacteria”, suggesting a rich area for future research with significant opportunities for potential discovery of novel endophytic microbes (Aghdam and Brown, 2021). This relatively high proportion of unclassified taxa likely reflects limitations of current reference databases, particularly for plant-associated endophytes, as well as the presence of novel or poorly characterized microbial lineages (Aghdam and Brown, 2021; Edgar, 2018; Thompson et al., 2017). Overall, these findings should be interpreted with several limitations. Due to the limited number of biological replicates per cultivar (≤4), the statistical support for comparisons is limited. As a result, post-hoc interpretations are tentative and will need further validation. Functional predictions were inferred using PICRUSt2 and were not validated by independent methods such as qPCR or cultivation-based assays. Future studies with larger sample sizes and experimental validation will be needed to confirm these findings. Additionally, this study does not directly assess plant health or disease outcomes, and therefore any links between microbiome composition and potential beneficial functions are not directly tested. How these differences in microbial diversity influence the health and growth of these plants warrants further research.

## Data Availability

The datasets generated for this study can be found in the NCBI Sequence Read Archive under accessions PRJNA1450107.
