# Supplementary material for: Diverse banana endophytes reveal potential genotype-driven community structure affected by domestication
Source: Front Microbiol. 2026 Jun 24;17:1830341. doi: 10.3389/fmicb.2026.1830341 (PMC13341605; doi:10.3389/fmicb.2026.1830341)
Supplement: Supplementary file 1 [file Supplementary_file_1.docx]

Supplementary Material

**Diverse banana endophytes reveal potential
genotype-driven community structure affected by domestication**

**Shiva A. Aghdam, Amanda M. V. Brown**

**Supplementary Table S1.** Banana samples, tissue type, and sample name were used in this study.

| **Banana cultivars** | **Genotype** | **Plant type** | **Year** | **Tissue** | **Sample ID** |
| --- | --- | --- | --- | --- | --- |
| *Musa balbisiana* | BB | Wild | 2019 | Below-ground | B1-C |
|  |  |  |  | Above-ground | B1-S |
|  |  |  | 2020 | Below-ground | B2-C |
|  |  |  |  | Above-ground | B2-S |
|  |  |  |  | Below-ground | B3-C |
|  |  |  |  | Above-ground | B3-S |
|  |  |  |  | Below-ground | B4-C |
|  |  |  |  | Above-ground | B4-S |
| Thai Black | BB | Wild | 2019 | Below-ground | BB1-C |
|  |  |  |  | Above-ground | BB1-S |
|  |  |  | 2020 | Below-ground | BB2-C |
|  |  |  |  | Above-ground | BB2-S |
|  |  |  |  | Below-ground | BB3-C |
|  |  |  |  | Above-ground | BB3-S |
|  |  |  |  | Below-ground | BB4-C |
|  |  |  |  | Above-ground | BB4-S |
| FHIA-25 | AAB | Domesticated | 2019 | Below-ground | F1-C |
|  |  |  |  | Above-ground | F1-S |
|  |  |  | 2020 | Below-ground | F2-C |
|  |  |  |  | Above-ground | F2-S |
|  |  |  |  | Below-ground | F3-C |
|  |  |  |  | Above-ground | F3-S |
| *Musa textilis* | TT | Wild | 2020 | Below-ground | T1-C |
|  |  |  |  | Above-ground | T1-S |
| Williams Hybrid | AAA | Domesticated | 2019 | Below-ground | W1-C |
|  |  |  |  | Above-ground | W1-S |
|  |  |  | 2020 | Below-ground | W2-C |
|  |  |  |  | Above-ground | W2-S |
|  |  |  |  | Below-ground | W3-C |
|  |  |  |  | Above-ground | W3-S |
|  |  |  |  | Below-ground | W4-C |
|  |  |  |  | Above-ground | W4-S |
| Dwarf Cavendish | AAA | Domesticated | 2019 | Below-ground | D1-C |
|  |  |  |  | Above-ground | D1-S |
|  |  |  | 2020 | Below-ground | D2-C |
|  |  |  |  | Above-ground | D2-S |
|  |  |  |  | Below-ground | D3-C |
|  |  |  |  | Above-ground | D3-S |
|  |  |  |  | Below-ground | D4-C |
|  |  |  |  | Above-ground | D4-S |

**Supplementary Table S2.** The relative resistance of these plants to various diseases, based on past studies.

| Cultivar / Species | Fusarium Wilt – RACE 1 | Fusarium Wilt – TR4 | Black Sigatoka | Banana Bunchy Top Virus | Banana Streak Virus | Moko (Ralstonia) | BXW (Xanthomonas wilt) |
| --- | --- | --- | --- | --- | --- | --- | --- |
| Dwarf Cavendish (AAA) | R [1] | S [2] | S | S [3] | S [4] | S [5] | S |
| Williams Hybrid (AAA) | R [2] | S [6] | S | S [7] | S [8] | S | S [9] |
| FHIA-25 (AAB Plantain) | R [10] | R [11] | R | S [12] | S | No study | S |
| *Musa balbisiana* (BB, wild) | R [13] | R [14] | R [15] | R [16] | S [17] | S [18] | R [19] |
| ‘Thai Black’ (BB, *Musa balbisiana* cultivar) | R  No study directly -BB genotype | R  No study directly -BB genotype [20] | No study | R  No study directly -BB genotype  [16] | S [21] | No study | R [19] |
| *Musa textilis* (Abacá) | S [22] | S [22] | No study | S [23] | No study | No study | No study |

* R = Resistant

* S = Susceptible

**Supplementary Table S3.** Sequencing outputs of bacterial endophytes from various banana cultivars.

| sample-id | input | filtered | percentage of input passed filter | denoised | merged | percentage of input merged | non-chimeric | percentage of input non-chimeric |
| --- | --- | --- | --- | --- | --- | --- | --- | --- |
| B1-S | 305520 | 252181 | 82.54 | 250057 | 238994 | 78.23 | 94636 | 30.98 |
| B2-S | 187291 | 154720 | 82.61 | 153334 | 145695 | 77.79 | 60472 | 32.29 |
| B3-S | 131376 | 109980 | 83.71 | 109240 | 106494 | 81.06 | 50827 | 38.69 |
| B4-S | 192235 | 162232 | 84.39 | 161693 | 159188 | 82.81 | 81834 | 42.57 |
| B1-C | 1406725 | 1081134 | 76.85 | 1073419 | 1028497 | 73.11 | 399909 | 28.43 |
| B2-C | 88574 | 54628 | 61.67 | 52594 | 48561 | 54.83 | 26006 | 29.36 |
| B3-C | 212881 | 183395 | 86.15 | 182011 | 175687 | 82.53 | 80840 | 37.97 |
| B4-C | 148522 | 123300 | 83.02 | 122542 | 120033 | 80.82 | 54904 | 36.97 |
| BB1-S | 115267 | 100785 | 87.44 | 99521 | 94371 | 81.87 | 46233 | 40.11 |
| BB2-S | 106551 | 92992 | 87.27 | 91815 | 86862 | 81.52 | 45193 | 42.41 |
| BB3-S | 86990 | 75873 | 87.22 | 75342 | 72647 | 83.51 | 34586 | 39.76 |
| BB4-S | 71240 | 64955 | 91.18 | 63817 | 61069 | 85.72 | 31946 | 44.84 |
| BB1-C | 319941 | 285078 | 89.1 | 282434 | 270060 | 84.41 | 108285 | 33.85 |
| BB2-C | 63208 | 57811 | 91.46 | 56503 | 51732 | 81.84 | 27005 | 42.72 |
| BB3-C | 83013 | 68536 | 82.56 | 67738 | 64490 | 77.69 | 29740 | 35.83 |
| BB4-C | 166125 | 124791 | 75.12 | 124292 | 121996 | 73.44 | 62914 | 37.87 |
| D1-S | 349989 | 312681 | 89.34 | 311749 | 307531 | 87.87 | 167088 | 47.74 |
| D2-S | 166659 | 153446 | 92.07 | 152657 | 148207 | 88.93 | 68295 | 40.98 |
| D3-S | 165661 | 144476 | 87.21 | 143754 | 140342 | 84.72 | 79820 | 48.18 |
| D4-S | 91301 | 78006 | 85.44 | 77428 | 75170 | 82.33 | 42694 | 46.76 |
| D1-C | 251343 | 236707 | 94.18 | 235303 | 230429 | 91.68 | 80027 | 31.84 |
| D2-C | 139737 | 123485 | 88.37 | 122804 | 120203 | 86.02 | 56962 | 40.76 |
| D3-C | 71818 | 64003 | 89.12 | 62055 | 55429 | 77.18 | 32776 | 45.64 |
| D4-C | 54671 | 47270 | 86.46 | 46923 | 46142 | 84.4 | 24856 | 45.46 |
| F1-S | 83345 | 76738 | 92.07 | 75323 | 69177 | 83 | 30486 | 36.58 |
| F2-S | 123132 | 112504 | 91.37 | 110290 | 100824 | 81.88 | 41628 | 33.81 |
| F3-S | 161743 | 140228 | 86.7 | 139455 | 136477 | 84.38 | 48861 | 30.21 |
| F1-C | 119445 | 110564 | 92.56 | 108229 | 99654 | 83.43 | 37140 | 31.09 |
| F2-C | 76924 | 70000 | 91 | 68349 | 61987 | 80.58 | 23969 | 31.16 |
| F3-C | 127461 | 115639 | 90.73 | 113455 | 105336 | 82.64 | 58114 | 45.59 |
| T1-S | 56731 | 52473 | 92.49 | 51245 | 45494 | 80.19 | 20119 | 35.46 |
| T1-C | 118444 | 88629 | 74.83 | 86134 | 76959 | 64.98 | 37447 | 31.62 |
| W1-S | 282330 | 196830 | 69.72 | 195476 | 177768 | 62.96 | 109306 | 38.72 |
| W2-S | 106196 | 85173 | 80.2 | 83277 | 77051 | 72.56 | 44414 | 41.82 |
| W3-S | 63309 | 56889 | 89.86 | 55702 | 50791 | 80.23 | 19786 | 31.25 |
| W4-S | 268743 | 160501 | 59.72 | 159404 | 155523 | 57.87 | 94157 | 35.04 |
| W1-C | 174367 | 143778 | 82.46 | 140080 | 128626 | 73.77 | 75992 | 43.58 |
| W2-C | 563342 | 408198 | 72.46 | 399907 | 369190 | 65.54 | 223021 | 39.59 |
| W3-C | 112733 | 92755 | 82.28 | 91141 | 84270 | 74.75 | 26955 | 23.91 |
| W4-C | 268211 | 240871 | 89.81 | 233086 | 183437 | 68.39 | 53056 | 19.78 |

**Supplementary Table S4.** Relative abundance of bacterial endophytes at family level from all cultivars.

| Family level | RA% |
| --- | --- |
| Proteobacteria;Gammaproteobacteria;Pseudomonadales;Pseudomonadaceae | 48.07345 |
| Unknown Bacteria | 27.21405 |
| Proteobacteria;Gammaproteobacteria;Enterobacterales;Enterobacteriaceae | 13.08625 |
| Proteobacteria;Alphaproteobacteria;Rhizobiales;Rhizobiaceae | 4.110278 |
| Proteobacteria;Gammaproteobacteria;Pseudomonadales;Moraxellaceae | 0.545046 |
| Proteobacteria;Alphaproteobacteria;Rhizobiales;Devosiaceae | 0.477438 |
| Proteobacteria;Gammaproteobacteria;Burkholderiales;Comamonadaceae | 0.458165 |
| Proteobacteria;Gammaproteobacteria;Burkholderiales;Oxalobacteraceae | 0.455768 |
| Proteobacteria;Gammaproteobacteria;Xanthomonadales;Xanthomonadaceae | 0.443073 |
| Bacteroidota;Bacteroidia;Chitinophagales;Chitinophagaceae | 0.344414 |
| Proteobacteria;Alphaproteobacteria;Sphingomonadales;Sphingomonadaceae | 0.311018 |
| Proteobacteria;Gammaproteobacteria;Enterobacterales;Pectobacteriaceae | 0.267832 |
| Proteobacteria;Alphaproteobacteria;Rhizobiales;Xanthobacteraceae | 0.265283 |
| Proteobacteria;Gammaproteobacteria;Enterobacterales;Erwiniaceae | 0.25585 |
| Proteobacteria;Gammaproteobacteria;Enterobacterales;__ | 0.246928 |
| Proteobacteria;Alphaproteobacteria;Caulobacterales;Caulobacteraceae | 0.231887 |
| Proteobacteria;Alphaproteobacteria;Rhizobiales;Pleomorphomonadaceae | 0.201652 |
| Firmicutes;Clostridia;Lachnospirales;Lachnospiraceae | 0.164992 |
| Proteobacteria;Gammaproteobacteria;Enterobacterales;Yersiniaceae | 0.146739 |
| Proteobacteria;Gammaproteobacteria;Aeromonadales;Aeromonadaceae | 0.125325 |
| Proteobacteria;Gammaproteobacteria;Burkholderiales;Rhodocyclaceae | 0.125325 |
| Proteobacteria;Alphaproteobacteria;Rhodobacterales;Rhodobacteraceae | 0.118595 |
| Proteobacteria;Gammaproteobacteria;Burkholderiales;Alcaligenaceae | 0.108601 |
| Actinobacteriota;Actinobacteria;Micrococcales;Cellulomonadaceae | 0.098149 |
| Actinobacteriota;Actinobacteria;Streptomycetales;Streptomycetaceae | 0.09147 |
| Proteobacteria;Gammaproteobacteria;Burkholderiales;Burkholderiaceae | 0.090807 |
| Proteobacteria;Gammaproteobacteria;Aeromonadales;__ | 0.089124 |
| Proteobacteria;Alphaproteobacteria;Acetobacterales;Acetobacteraceae | 0.084332 |
| Proteobacteria;Alphaproteobacteria;Rhizobiales;Beijerinckiaceae | 0.070004 |
| Proteobacteria;Alphaproteobacteria;Rickettsiales;Mitochondria | 0.068526 |
| Bacteroidota;Bacteroidia;Cytophagales;Microscillaceae | 0.063988 |
| Firmicutes;Bacilli;Paenibacillales;Paenibacillaceae | 0.062611 |
| Bacteroidota;Bacteroidia;Cytophagales;Spirosomaceae | 0.052108 |
| Actinobacteriota;Actinobacteria;Micrococcales;Microbacteriaceae | 0.047723 |
| Verrucomicrobiota;Verrucomicrobiae;Chthoniobacterales;Terrimicrobiaceae | 0.044409 |
| Proteobacteria;Gammaproteobacteria;Diplorickettsiales;Diplorickettsiaceae | 0.035334 |
| Proteobacteria;Gammaproteobacteria;Steroidobacterales;Steroidobacteraceae | 0.034722 |
| Proteobacteria;Gammaproteobacteria;Burkholderiales;Methylophilaceae | 0.029929 |
| Proteobacteria;Gammaproteobacteria;Xanthomonadales;Rhodanobacteraceae | 0.029725 |
| Cyanobacteria;Vampirivibrionia;Obscuribacterales;Obscuribacteraceae | 0.029674 |
| Proteobacteria;Alphaproteobacteria;Reyranellales;Reyranellaceae | 0.027788 |
| Firmicutes;Negativicutes;Veillonellales-Selenomonadales;Sporomusaceae | 0.027227 |
| Bacteroidota;Bacteroidia;Sphingobacteriales;Sphingobacteriaceae | 0.027176 |
| Actinobacteriota;Actinobacteria;Propionibacteriales;Propionibacteriaceae | 0.025136 |
| Proteobacteria;Alphaproteobacteria;Zavarziniales;Zavarziniaceae | 0.025034 |
| Proteobacteria;Gammaproteobacteria;Legionellales;Legionellaceae | 0.024932 |
| Proteobacteria;Alphaproteobacteria;Rhizobiales;Rhizobiales_Incertae_Sedis | 0.024728 |
| Verrucomicrobiota;Chlamydiae;Chlamydiales;cvE6 | 0.024066 |
| Verrucomicrobiota;Chlamydiae;Chlamydiales;Parachlamydiaceae | 0.023301 |
| Spirochaetota;Spirochaetia;Spirochaetales;Spirochaetaceae | 0.023046 |
| Proteobacteria;Alphaproteobacteria;Rhizobiales;Hyphomicrobiaceae | 0.022995 |
| Proteobacteria;Alphaproteobacteria;Rhizobiales;D05-2 | 0.021975 |
| Proteobacteria;Gammaproteobacteria;Gammaproteobacteria_Incertae_Sedis;Unknown_Family | 0.021108 |
| Bdellovibrionota;Oligoflexia;0319-6G20;0319-6G20 | 0.020853 |
| Proteobacteria;Alphaproteobacteria;Rhizobiales;Rhodomicrobiaceae | 0.019834 |
| Actinobacteriota;Actinobacteria;Corynebacteriales;Mycobacteriaceae | 0.019273 |
| Actinobacteriota;Actinobacteria;Pseudonocardiales;Pseudonocardiaceae | 0.019171 |
| Proteobacteria;Gammaproteobacteria;Enterobacterales;Hafniaceae | 0.018406 |
| Proteobacteria;Alphaproteobacteria;Azospirillales;Azospirillaceae | 0.018253 |
| Firmicutes;Bacilli;Bacillales;Bacillaceae | 0.018253 |
| Proteobacteria;__;__;__ | 0.017641 |
| Actinobacteriota;Actinobacteria;Micromonosporales;Micromonosporaceae | 0.017029 |
| Patescibacteria;Saccharimonadia;Saccharimonadales;LWQ8 | 0.0155 |
| Myxococcota;Polyangia;Polyangiales;BIrii41 | 0.014786 |
| Proteobacteria;Alphaproteobacteria;Rhizobiales;Kaistiaceae | 0.014684 |
| Verrucomicrobiota;Verrucomicrobiae;Verrucomicrobiales;Verrucomicrobiaceae | 0.01448 |
| Verrucomicrobiota;Verrucomicrobiae;Verrucomicrobiales;Rubritaleaceae | 0.013155 |
| Proteobacteria;Alphaproteobacteria;Rhizobiales;__ | 0.013104 |
| Proteobacteria;Gammaproteobacteria;__;__ | 0.013002 |
| Proteobacteria;Alphaproteobacteria;Rhizobiales;Ancalomicrobiaceae | 0.012237 |
| Proteobacteria;Alphaproteobacteria;__;__ | 0.011982 |
| Dependentiae;Babeliae;Babeliales;Vermiphilaceae | 0.011829 |
| Chloroflexi;Anaerolineae;SBR1031;A4b | 0.011829 |
| Bdellovibrionota;Bdellovibrionia;Bdellovibrionales;Bdellovibrionaceae | 0.011676 |
| Bacteroidota;Bacteroidia;Cytophagales;Cyclobacteriaceae | 0.011625 |
| Verrucomicrobiota;Verrucomicrobiae;Chthoniobacterales;Xiphinematobacteraceae | 0.010707 |
| Other | 0.531585 |

**Supplementary Table S5.** Pairwise differential abundance of bacterial endophytic families among banana cultivars, showing group comparisons, sample sizes (n1, n2), Z-statistics, raw and adjusted p-values, and significance levels (p < 0.05, p < 0.01).

| **Family** | **group1** | **group2** | **n1** | **n2** | **stat_z** | **p_raw** | **p_adj** | **p_adj_signif** |
| --- | --- | --- | --- | --- | --- | --- | --- | --- |
| *Devosiaceae* | Dwarf Cavendish | Williams Hybrid | 8 | 8 | 3.849295 | 0.000118 | 0.001777 | ** |
| *Lachnospiraceae* | Dwarf Cavendish | Williams Hybrid | 8 | 8 | 3.464366 | 0.000531 | 0.007972 | ** |
| *Rhodobacteraceae* | Dwarf Cavendish | Williams Hybrid | 8 | 8 | 3.378826 | 0.000728 | 0.010919 | * |
| *Pseudomonadaceae* | Thai Black | Williams Hybrid | 8 | 8 | -3.05805 | 0.002228 | 0.011139 | * |
| *Pseudomonadaceae* | Dwarf Cavendish | Williams Hybrid | 8 | 8 | -3.35744 | 0.000787 | 0.011139 | * |
| *Pseudomonadaceae* | *Musa balbisiana* | Williams Hybrid | 8 | 8 | -3.12221 | 0.001795 | 0.011139 | * |
| *Rhodobacteraceae* | Dwarf Cavendish | *Musa textilis* | 8 | 2 | 3.11076 | 0.001866 | 0.013995 | * |
| *Devosiaceae* | Thai Black | Williams Hybrid | 8 | 8 | 2.951126 | 0.003166 | 0.015831 | * |
| *Devosiaceae* | *Musa balbisiana* | Williams Hybrid | 8 | 8 | 3.015281 | 0.002567 | 0.015831 | * |
| *Enterobacteriaceae* | Thai Black | *Musa balbisiana* | 8 | 8 | 2.951126 | 0.003166 | 0.015831 | * |
| *Enterobacteriaceae* | Dwarf Cavendish | *Musa balbisiana* | 8 | 8 | 3.015281 | 0.002567 | 0.015831 | * |
| *Enterobacteriaceae* | FHIA-25 | *Musa balbisiana* | 6 | 8 | 3.035791 | 0.002399 | 0.015831 | * |
| *Rhodocyclaceae* | Dwarf Cavendish | Williams Hybrid | 8 | 8 | 3.250516 | 0.001152 | 0.017279 | * |
| *Pectobacteriaceae* | Dwarf Cavendish | Williams Hybrid | 8 | 8 | 3.207746 | 0.001338 | 0.020067 | * |
| *Rhodobacteraceae* | Thai Black | Dwarf Cavendish | 8 | 8 | -2.8442 | 0.004452 | 0.022261 | * |
| *Spirosomaceae* | FHIA-25 | *Musa balbisiana* | 6 | 8 | -3.12159 | 0.001799 | 0.026982 | * |
| *Lachnospiraceae* | Dwarf Cavendish | *Musa textilis* | 8 | 2 | 2.89436 | 0.003799 | 0.028495 | * |
| *Alcaligenaceae* | *Musa balbisiana* | Williams Hybrid | 8 | 8 | 3.058051 | 0.002228 | 0.033417 | * |
| *Sphingobacteriaceae* | FHIA-25 | *Musa balbisiana* | 6 | 8 | -2.79161 | 0.005245 | 0.039335 | * |
| *Sphingobacteriaceae* | *Musa balbisiana* | Williams Hybrid | 8 | 8 | 2.993896 | 0.002754 | 0.039335 | * |
| *Comamonadaceae* | Dwarf Cavendish | Williams Hybrid | 8 | 8 | 2.993896 | 0.002754 | 0.041316 | * |
| *Zavarziniaceae* | Thai Black | *Musa textilis* | 8 | 2 | 2.813209 | 0.004905 | 0.041703 | * |
| *Zavarziniaceae* | *Musa balbisiana* | *Musa textilis* | 8 | 2 | 2.772634 | 0.00556 | 0.041703 | * |
| *Devosiaceae* | Dwarf Cavendish | *Musa textilis* | 8 | 2 | 2.515658 | 0.011881 | 0.044554 | * |

**Supplementary Table S6.** Pairwise differential abundance of bacterial endophytic genera among banana cultivars, showing group comparisons, sample sizes (n1, n2), Z-statistics, raw and adjusted p-values, and significance levels (p < 0.05, p < 0.01).

| **Genus** | **group1** | **group2** | **n1** | **n2** | **stat_z** | **p_raw** | **p_adj** | **p_adj_signif** |
| --- | --- | --- | --- | --- | --- | --- | --- | --- |
| *Acidovorax* | Dwarf Cavendish | Williams Hybrid | 8 | 8 | 3.293286 | 0.00099 | 0.014854 | * |
| *Devosia* | Dwarf Cavendish | Williams Hybrid | 8 | 8 | 3.87068 | 0.000109 | 0.001628 | ** |
| *Devosia* | Thai Black | Williams Hybrid | 8 | 8 | 3.015281 | 0.002567 | 0.013772 | * |
| *Devosia* | *Musa balbisiana* | Williams Hybrid | 8 | 8 | 2.993896 | 0.002754 | 0.013772 | * |
| *Devosia* | Dwarf Cavendish | *Musa textilis* | 8 | 2 | 2.529183 | 0.011433 | 0.042873 | * |
| *Kosakonia* | FHIA-25 | *Musa balbisiana* | 6 | 8 | 3.108386 | 0.001881 | 0.028217 | * |
| *Kosakonia* | Dwarf Cavendish | *Musa balbisiana* | 8 | 8 | 2.801431 | 0.005088 | 0.038157 | * |
| *Kosakonia* | FHIA-25 | Williams Hybrid | 6 | 8 | 2.633219 | 0.008458 | 0.03968 | * |
| *Kosakonia* | *Musa balbisiana* | *Musa textilis* | 8 | 2 | -2.55623 | 0.010581 | 0.03968 | * |
| *Lachnotalea* | Dwarf Cavendish | Williams Hybrid | 8 | 8 | 3.122206 | 0.001795 | 0.026925 | * |
| *Oxalicibacterium* | Thai Black | Williams Hybrid | 8 | 8 | 2.993896 | 0.002754 | 0.013772 | * |
| *Oxalicibacterium* | Dwarf Cavendish | Williams Hybrid | 8 | 8 | 3.079436 | 0.002074 | 0.013772 | * |
| *Oxalicibacterium* | *Musa balbisiana* | Williams Hybrid | 8 | 8 | 3.186361 | 0.001441 | 0.013772 | * |
| *Pectobacterium* | Thai Black | *Musa balbisiana* | 8 | 8 | 2.801431 | 0.005088 | 0.038157 | * |
| *Pectobacterium* | Thai Black | Williams Hybrid | 8 | 8 | 2.822816 | 0.00476 | 0.038157 | * |
| *Pseudomonas* | Dwarf Cavendish | Williams Hybrid | 8 | 8 | -3.22913 | 0.001242 | 0.010033 | * |
| *Pseudomonas* | *Musa balbisiana* | Williams Hybrid | 8 | 8 | -3.20775 | 0.001338 | 0.010033 | * |
| *Pseudomonas* | Thai Black | Williams Hybrid | 8 | 8 | -3.01528 | 0.002567 | 0.012837 | * |
| *Raoultella* | *Musa balbisiana* | Williams Hybrid | 8 | 8 | -3.25052 | 0.001152 | 0.017279 | * |
| *Sediminibacterium* | Dwarf Cavendish | *Musa balbisiana* | 8 | 8 | 3.293286 | 0.00099 | 0.007427 | ** |
| *Sediminibacterium* | *Musa balbisiana* | Williams Hybrid | 8 | 8 | -3.29329 | 0.00099 | 0.007427 | ** |
| *Sphingopyxis* | Thai Black | Williams Hybrid | 8 | 8 | 3.122206 | 0.001795 | 0.026925 | * |
| *Sphingopyxis* | FHIA-25 | Williams Hybrid | 6 | 8 | 2.831205 | 0.004637 | 0.03478 | * |
| *Uliginosibacterium* | FHIA-25 | Williams Hybrid | 6 | 8 | 3.385567 | 0.00071 | 0.010655 | * |
| *Uliginosibacterium* | Dwarf Cavendish | Williams Hybrid | 8 | 8 | 3.122206 | 0.001795 | 0.013463 | * |
| *Xylella* | Dwarf Cavendish | Williams Hybrid | 8 | 8 | 3.036666 | 0.002392 | 0.035882 | * |

**Supplementary Figure S1.** Rarefaction curves of banana endophytic microbiomes. Observed ASVs were plotted against sequencing depth for each sample.


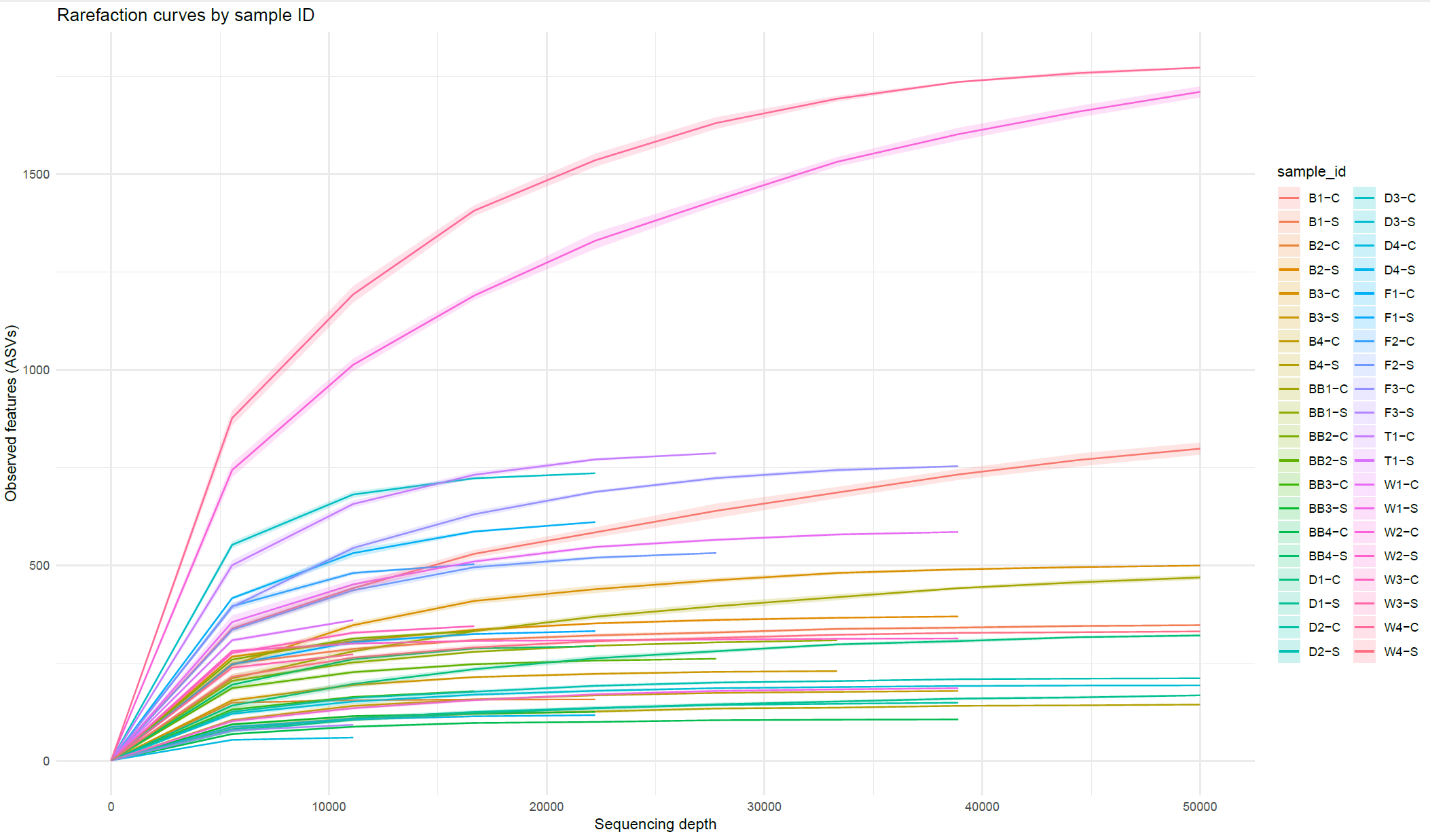


**Supplementary Figure S2.** Phylogenetic tree depicting the endophytic microbiomes of banana plants. The phylogenetic tree was generated using QIIME and drawn with q2-empress. The phylogeny is colored by phyla with the most abundant phyla. Tree branches and the first innermost layer are colored at the phylum level. The outermost layer shows the distribution of ASVs at the family level within the dominant phyla.


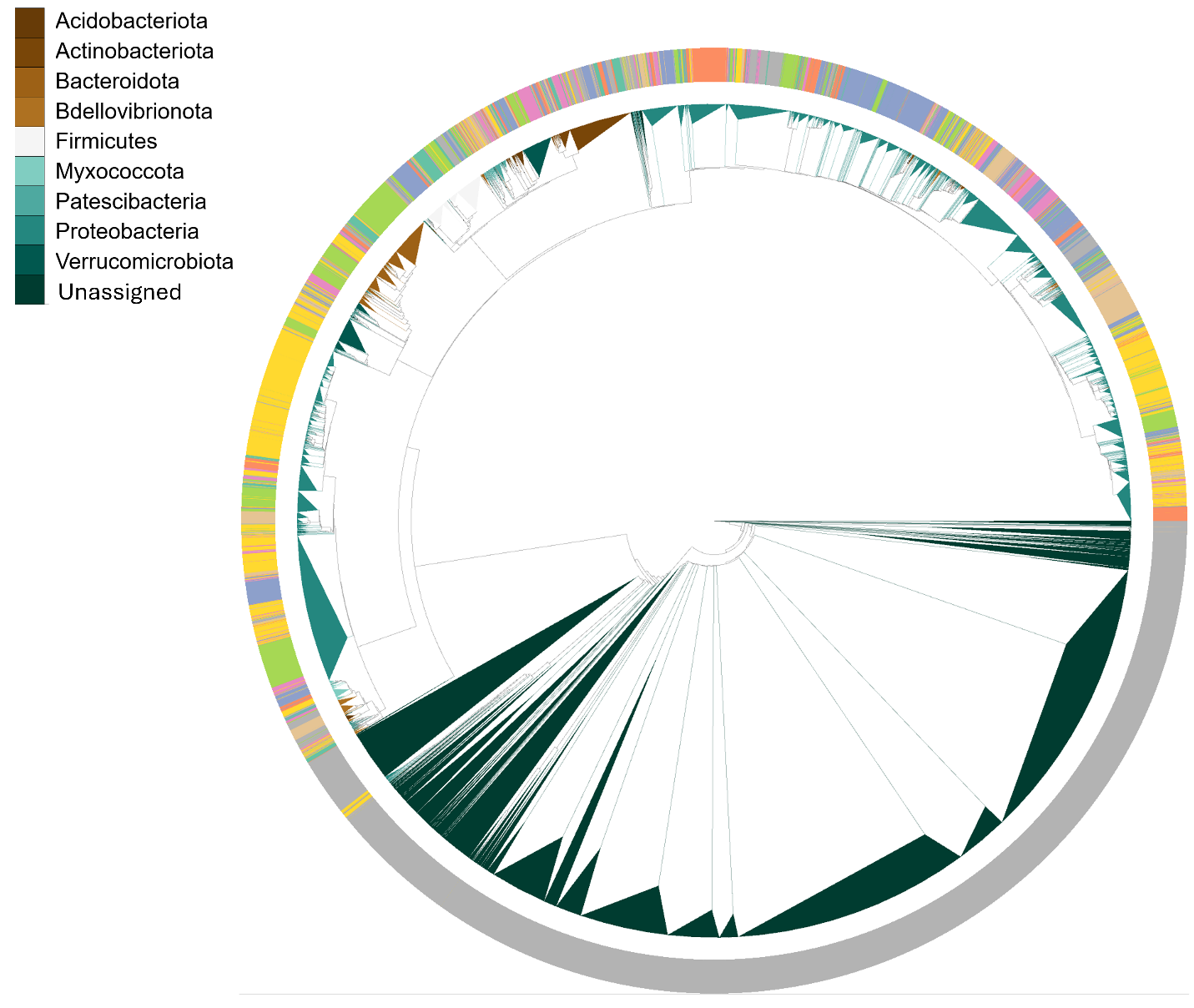


**Supplementary Figure S3.** Comparative analysis of individual ASVs at the family level across banana genotypes. The box plots show the log-transformed abundance (log10(abundance+1)) of ASVs assigned to specific bacterial families, including *Pseudomonadaceae*, *Enterobacteriaceae*, *Rhizobiaceae*, *Devosiaceae*, *Xanthomonadaceae*, *Comamonadaceae*, *Oxalobacteraceae*, and *Sphingomonadaceae*. Each box represents the variation in ASV abundance among the six genotypes: MB (*Musa balbisiana*), BB (Thai Black), MT (*Musa textilis*), DC (Dwarf Cavendish), WH (Williams Hybrid), and FH (FHIA-25). This analysis highlights genotype-specific differences in microbiome composition at the family level.

**
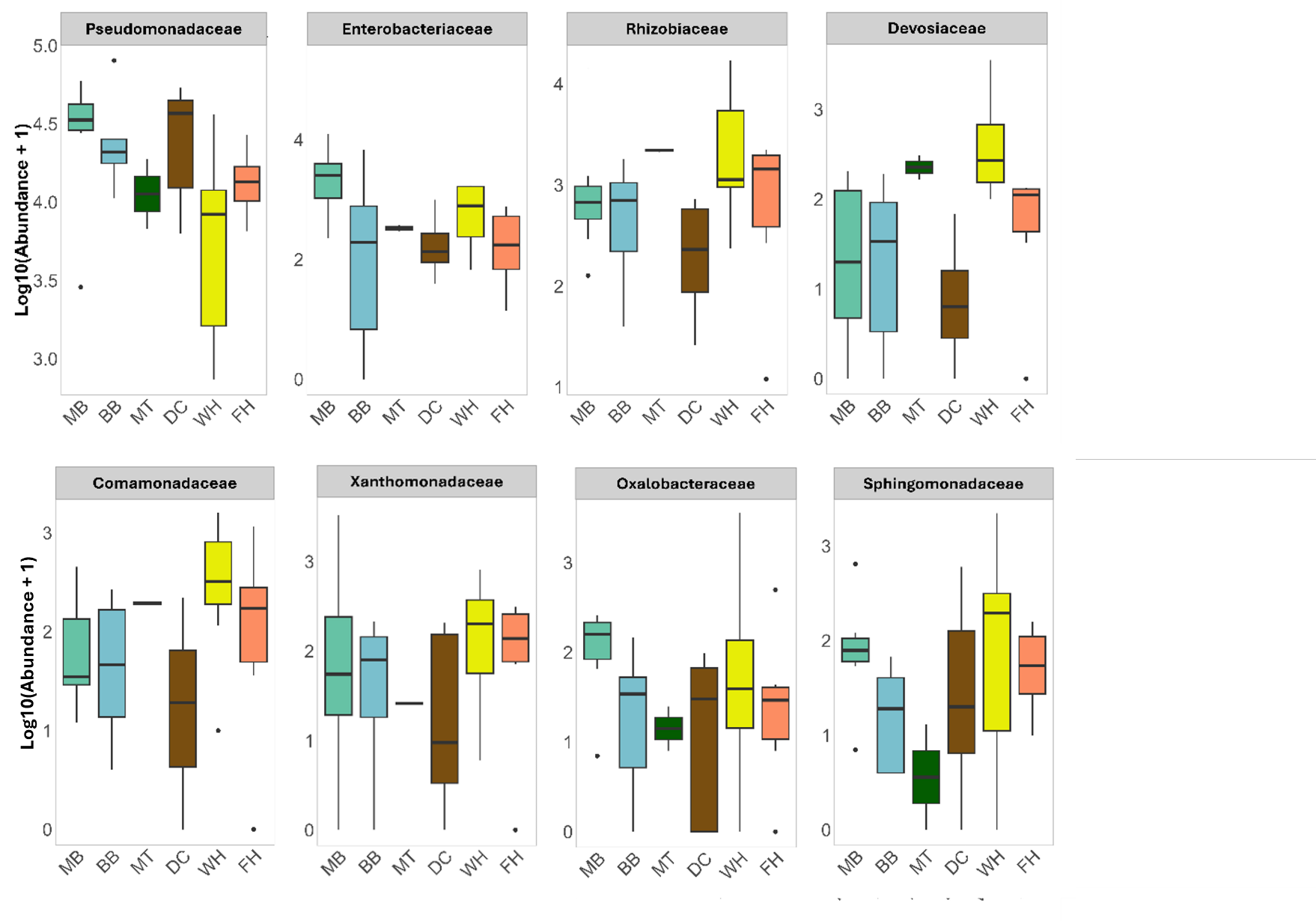
**

**Supplementary Figure** **S4.** Overlap of taxa at the genus level among all banana cultivars in wild and domesticated plants from (A) above-ground and (B) below-ground tissue. Below-ground tissues hosted 647 bacterial genera, with Williams Hybrid harboring the highest number of unique genera (168), while Thai Black showed the fewest (14), with domesticated plants sharing 109 genera and wild-type plants sharing 64 genera. In above-ground tissues, 339 genera were detected, with domesticated plants sharing 39 genera compared to only 10 in wild plants.

1. Above ground


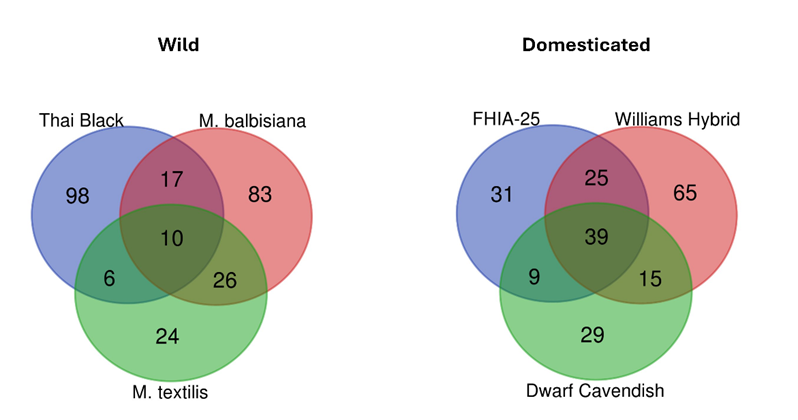


1. Below-ground


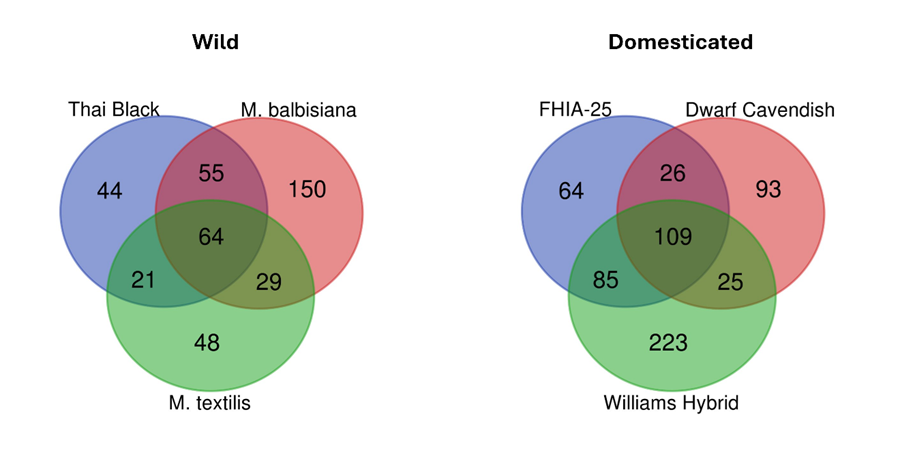


**Supplementary Figure S5.** Alpha diversity compares microbiomes in wild and domesticated banana cultivars.


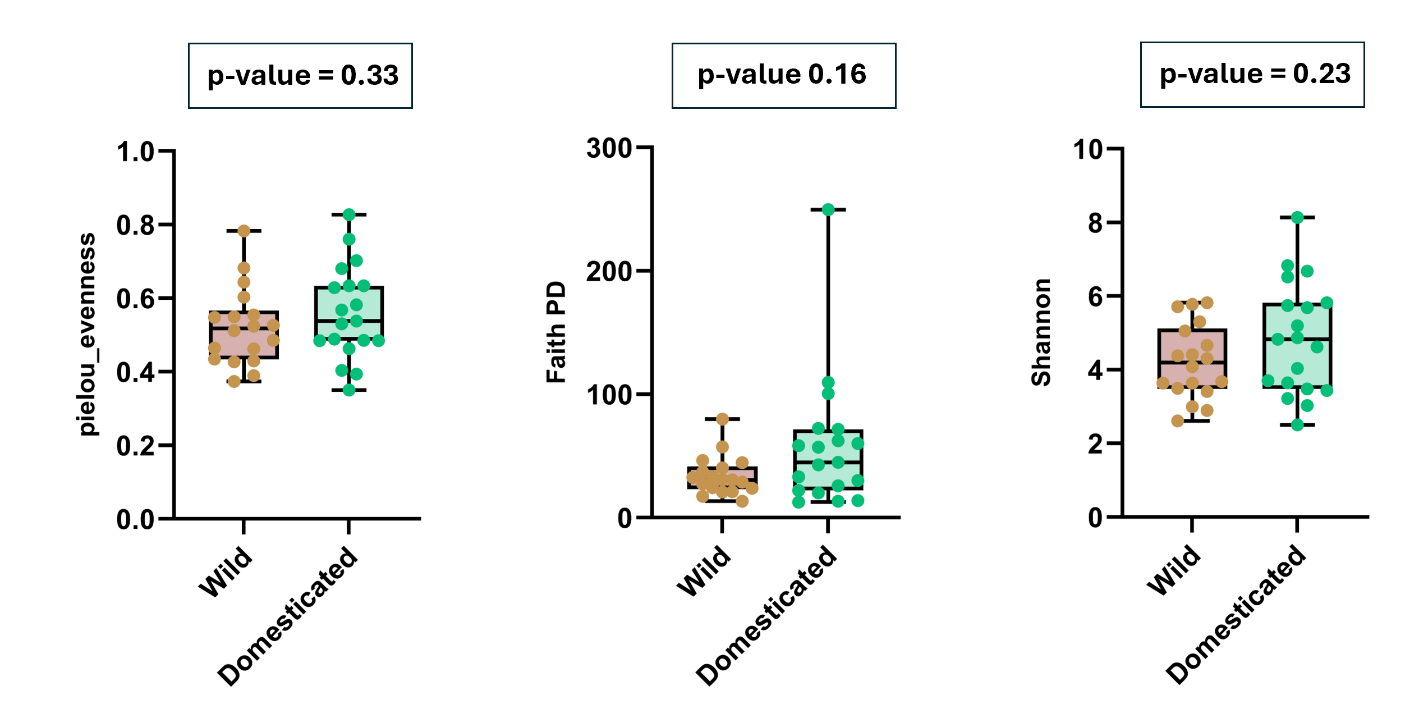


**Supplementary Figure S6.** Beta diversity of endophytic microbiota among above and below-ground tissues using Unweighted and Weighted UniFrac metrics shown by PCoA plots.


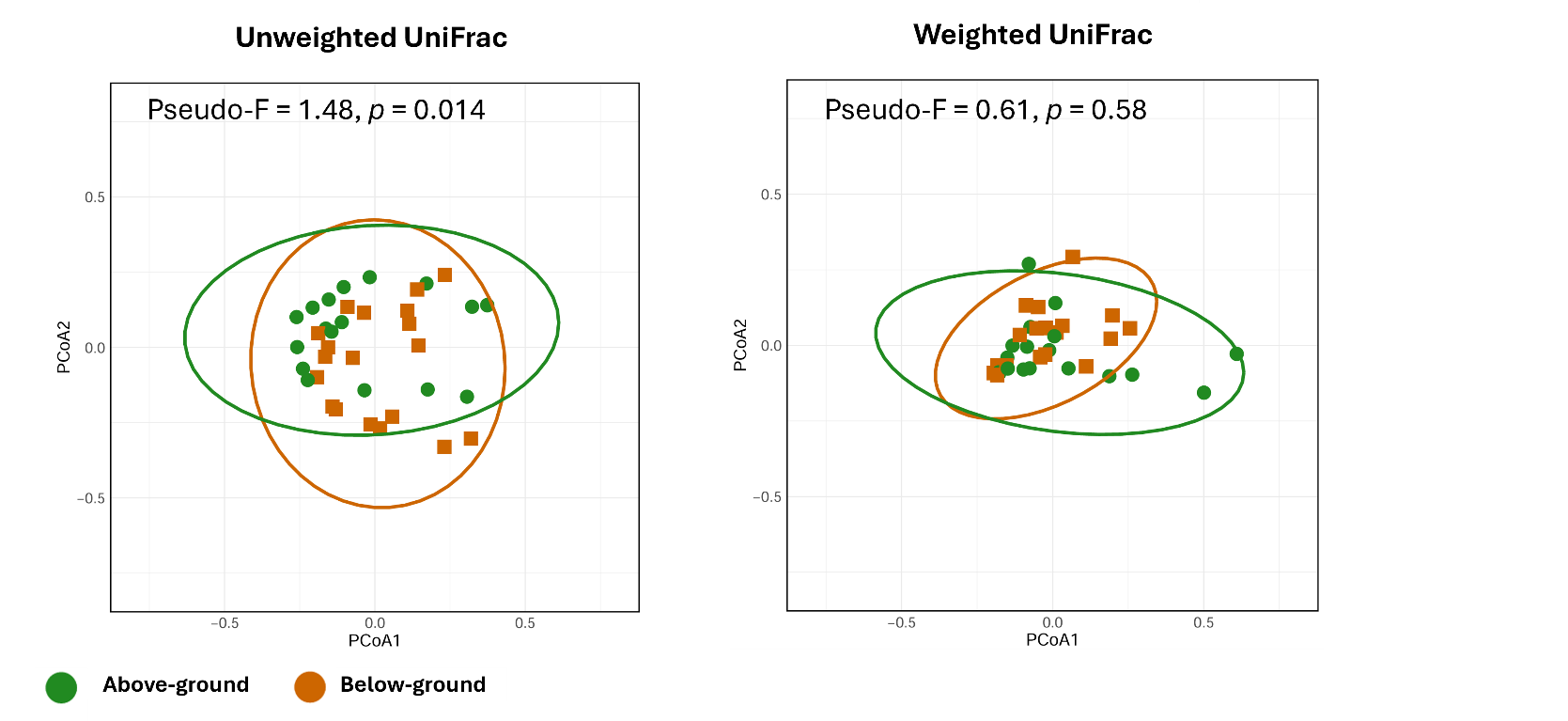


**Supplementary Figure S7.** Beta diversity of endophytic microbiota among wild-type and domesticated banana cultivars using Unweighted and Weighted UniFrac metrics shown by PCoA plots.


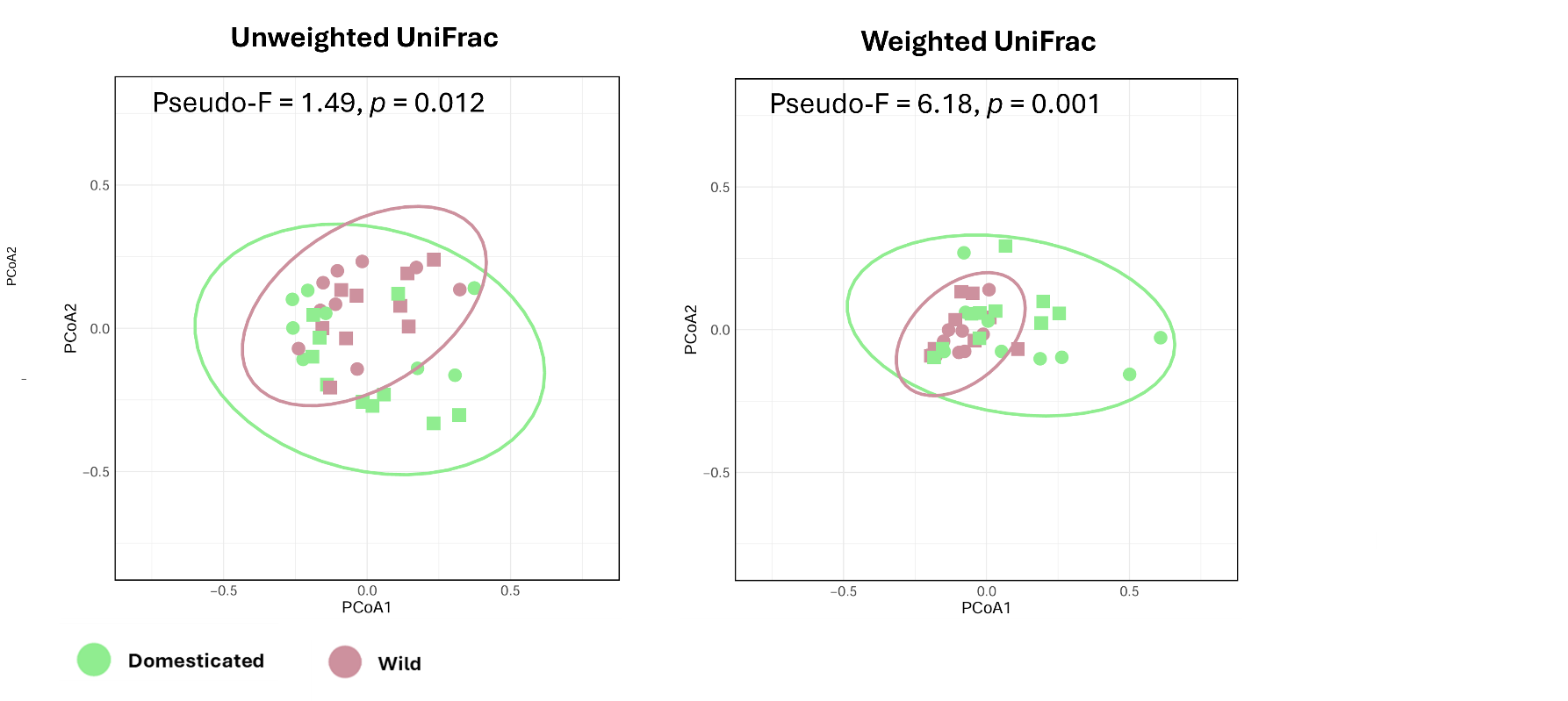


References:

1. Thangavelu, R., et al., *Screening of exotic banana accessions for their resistance to fusarium wilt race 1 and tropical race 4 in India.* Scientific Reports, 2025. **15**(1): p. 25060.

2. Dale, J., et al., *Transgenic Cavendish bananas with resistance to Fusarium wilt tropical race 4.* Nature Communications, 2017. **8**(1): p. 1496.

3. Bashir, S., et al., *Quantitation of Multipartite Banana Bunchy Top Virus Genomic Components and Their Transcripts in Infected Tissues of Banana (Musa acuminata).* Agronomy, 2022. **12**(12).

4. James, A.P., et al., *Infectivity of an Infectious Clone of Banana Streak CA Virus in A-Genome Bananas (Musa acuminata ssp.).* Viruses, 2021. **13**(6).

5. Blomme, G., et al., *Bacterial Diseases of Bananas and Enset: Current State of Knowledge and Integrated Approaches Toward Sustainable Management.* Frontiers in Plant Science, 2017. **Volume 8 - 2017**.

6. Fei, S., et al., *Small RNA profiling of Cavendish banana roots inoculated with Fusarium oxysporum f. sp. cubense race 1 and tropical race 4.* Phytopathology Research, 2019. **1**(1): p. 22.

7. Hooks, C.R., et al., *Comparative susceptibility of two banana cultivars to Banana bunchy top virus under laboratory and field environments.* J Econ Entomol, 2009. **102**(3): p. 897–904.

8. Geering, A.D., et al., *Analysis of the distribution and structure of integrated Banana streak virus DNA in a range of Musa cultivars.* Mol Plant Pathol, 2001. **2**(4): p. 207–13.

9. Marín-Ortiz, J.C., et al., *Early detection of bacterial wilt in bananas caused by Ralstonia solanacearum using reflectance spectroscopy.* Journal of Plant Diseases and Protection, 2023. **131**(2): p. 523–531.

10. Gaidashova, S.V., F. Karemera, and E.B. Karamura, *Agronomic performance of introduced banana varieties in lowlands of Rwanda.* African Crop Science Journal, 2010. **16**(1).

11. Chen, A., et al., *Assessing Variations in Host Resistance to Fusarium oxysporum f sp. Cubense Race 4 in Musa Species, with a Focus on the Subtropical Race 4.* Frontiers in Microbiology, 2019. **10**(MAY).

12. Chabi, M., et al., *Variation in Symptom Development and Infectivity of Banana Bunchy Top Disease among Four Cultivars of Musa sp.* Crops, 2023. **3**(2): p. 158–169.

13. Thangavelu, R., et al., *Identification of sources resistant to a virulent Fusarium wilt strain (VCG 0124) infecting Cavendish bananas.* Scientific Reports, 2021. **11**(1): p. 3183.

14. Li, W.M., et al., *Resistance sources to Fusarium oxysporum f. sp. cubense tropical race 4 in banana wild relatives.* Plant Pathology, 2015. **64**(5): p. 1061–1067.

15. Kimunye, J., et al., *Sources of resistance to Pseudocercospora fijiensis, the cause of black Sigatoka in banana.* Plant Pathol, 2021. **70**(7): p. 1651–1664.

16. Dela Cueva, F.M., et al., *Resistance of Musa balbisiana Accessions of the Philippines to Banana Bunchy Top Virus.* Plant Dis, 2023. **107**(7): p. 1973–1978.

17. Gayral, P., et al., *A Single Banana Streak Virus Integration Event in the Banana Genome as the Origin of Infectious Endogenous Pararetrovirus.* Journal of Virology, 2008. **82**(13): p. 6697–6710.

18. Blomme, G., et al., *Bacterial diseases of bananas and enset: Current state of knowledge and integrated approaches toward sustainable management.* Frontiers in Plant Science, 2017. **8**(July): p. 1–25.

19. Tripathi, L., et al., *Molecular Basis of Disease Resistance in Banana Progenitor Musa balbisiana against Xanthomonas campestris pv. musacearum.* Scientific Reports, 2019. **9**(1): p. 7007.

20. Zuo, C., et al., *Germplasm screening of Musa spp. For resistance to Fusarium oxysporum f. sp. cubense tropical race 4 (Foc TR4).* European Journal of Plant Pathology, 2018. **151**(3): p. 723–734.

21. Tripathi, J.N., et al., *CRISPR/Cas9 editing of endogenous banana streak virus in the B genome of Musa spp. overcomes a major challenge in banana breeding.* Communications Biology, 2019. **2**(1): p. 46.

22. Martínez-de la Parte, E., et al., *Tropical Race 4 and Race 1 strains causing Fusarium wilt of banana infect and survive in Heliconia species and ornamental bananas.* European Journal of Plant Pathology, 2025. **171**(2): p. 157–166.

23. Parac, E.P. and A.G. Lalusin. *Resistance Reaction of Abaca ( Musa textilis Nee) Hybrids to Bunchy Top and Establishment of Disease Severity Rating Scale for Screenhouse Screening*. 2022.
